# The mechanosensitive adhesion G protein-coupled receptor 133 (GPR133/ADGRD1) enhances bone formation

**DOI:** 10.1038/s41392-025-02291-y

**Published:** 2025-06-30

**Authors:** Juliane Lehmann, Hui Lin, Zihao Zhang, Maren Wiermann, Albert M. Ricken, Franziska Brinkmann, Jana Brendler, Christian Ullmann, Luisa Bayer, Sandra Berndt, Anja Penk, Nadine Winkler, Franz Wolfgang Hirsch, Thomas Fuhs, Josef Käs, Peng Xiao, Torsten Schöneberg, Martina Rauner, Jin-Peng Sun, Ines Liebscher

**Affiliations:** 1 Rudolf Schönheimer Institute of Biochemistry, Medical Faculty, University of Leipzig, Leipzig, Germany; 2 Department of Biochemistry and Molecular Biology, School of Basic Medical Sciences, Cheeloo College of Medicine, Shandong University, Jinan, Shandong China; 3 New Cornerstone Science Laboratory, Shandong University, Jinan, China; 4 Key Laboratory Experimental Teratology of the Ministry of Education and Department of Physiology, School of Basic Medical Sciences, Shandong University, Jinan, Shandong China; 5 Institute of Anatomy, Medical Faculty, Leipzig University, Leipzig, Germany; 6 Department of Medicine III & Center for Healthy Aging, Medical Faculty and University Hospital Carl Gustav Carus, Dresden University of Technology, Dresden, Germany; 7 Institute for Medical Physics and Biophysics, University of Leipzig, Leipzig, Germany; 8 Department of Pediatric Radiology, University of Leipzig, Leipzig, Germany; 9 Soft Matter Physics Division, Peter Debye Institute for Soft Matter Physics, University of Leipzig, Leipzig, Germany

**Keywords:** Endocrine system and metabolic diseases, Molecular medicine, Endocrine system and metabolic diseases, Physiology, Cell biology

## Abstract

Osteoporosis represents an increasing health and socioeconomic burden on aging societies. Current therapeutic options often come with potentially severe side effects or lack long-term efficacy, highlighting the urgent need for more effective treatments. Identifying novel drug targets requires a thorough understanding of their physiological roles. Genome-wide association studies in humans have linked gene variants of the adhesion G protein-coupled receptor 133 (GPR133/ADGRD1) to variations in bone mineral density and body height. In this study, we explore the impact of GPR133/ADGRD1 on osteoblast differentiation and function. Constitutive and osteoblast-specific knockouts of *Gpr133/Adgrd1* in mice lead to reduced cortical bone mass and trabecularization in the femurs and vertebrae — features characteristic of osteoporosis. This osteopenic phenotype in receptor-deficient mice is caused by impaired osteoblast function, which, in turn, promotes increased osteoclast activity. At the molecular level, GPR133/ADGRD1 regulates osteoblast function and differentiation through a combined activation mechanism involving interaction with its endogenous ligand, protein tyrosine kinase 7 (PTK7), and mechanical forces. This is demonstrated in vitro through stretch assays and in vivo via a mechanical loading experiment. Further in vitro analysis shows that GPR133/ADGRD1-mediated osteoblast differentiation is driven by cAMP-dependent activation of the β-catenin signaling pathway. Activation of GPR133/ADGRD1 with the receptor-specific ligand AP-970/43482503 (AP503) enhances osteoblast function and differentiation, both in vitro and in vivo, significantly alleviating osteoporosis in a mouse ovariectomy model. These findings position GPR133/ADGRD1 as a promising therapeutic target for osteoporosis and other diseases characterized by reduced bone mass.

## Introduction

Osteoporosis is a skeletal disorder characterized by low bone mineral density and altered bone microstructure, which predispose individuals to low-impact fragility fractures.^1^ In general, osteoporosis results from an imbalance between bone formation and bone resorption, a process intricately regulated by the coordinated actions of bone-forming osteoblasts and bone-resorbing osteoclasts (reviewed in ref. ^2^). Osteoblasts originate from multipotent mesenchymal stromal cells (MSCs), and their differentiation is driven by various growth factors and cytokines. These signaling molecules trigger the expression of key transcription factors and osteoblast-specific genes such as runt-related transcription factor 2 (RUNX2), osterix (OSX), alkaline phosphatase (ALP), and osteocalcin (OCN). The primary role of osteoblasts is to synthesize the bone matrix by producing type I collagen and a range of non-collagenous proteins, including OCN, osteonectin, osteopontin, and proteoglycans. Osteoblasts initiate bone formation by generating an unmineralized organic matrix called osteoid, which later undergoes mineralization through the deposition of hydroxyapatite crystals—also produced by osteoblasts. During the mineralization phase, osteoblasts exhibit elevated ALP activity, making ALP a widely recognized marker for osteoblast activity and maturation. Beyond their bone-forming function, osteoblasts also regulate osteoclastogenesis by secreting receptor activator of nuclear factor-κB ligand (RANKL) and macrophage colony-stimulating factor (M-CSF). Once embedded within the bone matrix, osteoblasts can transition into osteocytes, which act as mechanosensors within the bone matrix, facilitating the interaction and differentiation of both osteoblasts and osteoclasts.

Fractures due to osteoporosis significantly reduce quality of life, increasing morbidity, disability, and mortality.^3,4^ According to the International Osteoporosis Foundation, approximately 25.5 million women and 6.5 million men aged 50+ were estimated to suffer from osteoporosis in the EU in 2019, with more than 3.2 million new fragility fractures occurring each year.^1^ Beyond the clinical burden, the economic impact of osteoporosis is substantial, with costs projected to exceed $25 billion annually to treat over three million fractures in the United States by 2025.^5^ Osteoporosis is often considered a “silent” disease, as it typically presents no symptoms until a fracture occurs. In addition to primary osteoporosis, which is related to aging and declining sex hormones, secondary osteoporosis can result from immobilization (e.g., after surgery), diseases (e.g., cancer), or treatments (e.g., glucocorticoids). While a genetic contribution to osteoporosis has been recognized, single-gene causes are rarely identified, and polygenic linkages remain functionally unvalidated.^6^

Current therapies often focus on vitamin D and/or calcium supplementation, and more specific pharmacological treatments are associated with severe side effects.^7–9^ For example, postmenopausal estrogen therapy increases the risk of cancer and thrombosis, while parathyroid hormone therapy offers benefits only within a limited two-year window before its catabolic effects emerge. To develop new therapies, understanding the physiological roles and mechanisms of potential targets is essential. A promising target is the orphan G protein-coupled receptor (GPCR), GPR133 (also known as ADGRD1), which has been identified as a potential causal genetic driver of bone mineral density (BMD) in humans,^10^ and its genetic variants are associated with differences in human body height^11^ (for more details, see supplementary Text). Despite this, its exact role in bone homeostasis remains unclear. Given that several functionally significant single nucleotide polymorphisms (SNPs) have been identified in human populations,^12^ understanding GPR133/ADGRD1’s role in bone formation and mineralization is of high interest for preventing and treating both receptor deficiency-related and receptor-independent (e.g., postmenopausal) osteoporosis. Given that the GPCR class is among the most widely used molecular targets for therapeutics approved by the U.S. Food and Drug Administration, GPR133/ADGRD1 could serve as a new drug target for treating bone disorders. GPR133/ADGRD1 is an adhesion G protein-coupled receptor (aGPCR) that exhibits typical characteristics of this receptor class, including a complex N-terminus with a GPCR autoproteolysis-inducing (GAIN)^13^ and pentraxin domain. The receptor signals through the intracellular G_s_/adenylyl cyclase/cAMP pathway.^14^ Its activation depends on an internal agonistic *Stachel* sequence,^15^ which is likely exposed by mechanical forces^16,17^ or interaction with its extracellular ligands, protein tyrosine kinase 7 (PTK7)^18^ and plexin domain-containing protein 2 (Plxdc2).^19^ In a recent study we discovered α-dihydrotestosterone as agonist of GPR133/ADGRD1 and identified the first specific and highly potent small molecule agonist, AP-970/43482503 (AP503), for the receptor.^20^

In line with previous human association studies, we demonstrate that constitutive *Gpr133/Adgrd1*-deficient mice exhibit reduced bone volume and strength. A lower number of osteoblasts and a reduced bone-formation rate suggest a specific role for GPR133/ADGRD1 in osteoblast function. Phenotypic replication of constitutive knockout (KO) findings in an osteoblast precursor-specific *Gpr133/Adgrd1* KO mouse line, as well as in vitro assays showing reduced osteoblast differentiation and function under receptor knockdown (KD) conditions, further support the role of GPR133/ADGRD1 in osteoblasts. GPR133/ADGRD1 activation in osteoblasts depends on a combination of mechanical force and PTK7 interaction, which induces cAMP-dependent activation of the β-catenin pathway. The recently developed GPR133/ADGRD1 agonist AP503^20^ induces osteoblastogenesis in vitro and in vivo and significantly alleviates osteoporosis in vivo in an ovariectomy mouse model.

## Results

### *Gpr133/Adgrd1* is expressed in mouse bone and its absence affects body length, bone thickness and bone mineral density

**Fig. 1:**
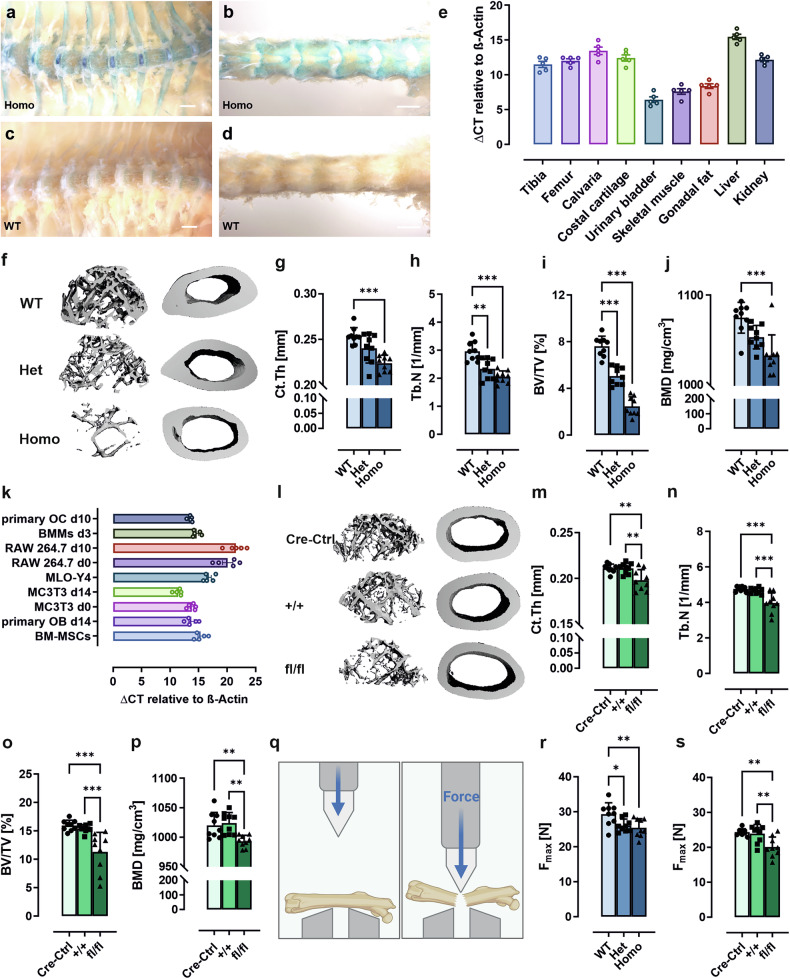
Constitutive *Gpr133/Adgrd1* deficiency in male mice results in trabecular and cortical bone loss. **a–d** LacZ staining of the entire spine of 3-months-old male *Gpr133/Adgrd1* homozygous (Homo) knockout (KO) and wild-type (WT) mice. Scale bar: 1 mm (a/c) and 2 mm (b/d). **e** *Gpr133/Adgrd1* mRNA expression in different bone and organs of 23-weeks-old male WT mice using qPCR (*n* = 5). **f–j** Femora from 23-weeks-old male WT, heterozygous (Het), or homozygous (Homo) *Gpr133/Adgrd1* KO mice were examined by µCT. **f** Representative 3D reconstructions of the trabecular compartment and the femoral midshaft. **g** Cortical thickness (Ct.Th), (**h**) trabecular number (Tb.N), (**i**) bone volume/total volume (BV/TV) and (**j**) cortical bone mineral density (BMD) were measured at the femoral midshaft. (*n* = 9 per group). **k** Basal *Gpr133/Adgrd1* mRNA expression levels determined by qPCR (ΔCT) in undifferentiated bone marrow-derived mesenchymal stromal cells (BM-MSCs), differentiated primary osteoblasts (OBs), MC3T3 cells (osteoblast-like cell line), MLO-Y4 cells (osteocyte-like cell line), RAW264.7 cells (osteoclast-like cell line), undifferentiated bone marrow macrophages (BMMs), and differentiated primary osteoclasts (OCs). (*n* = 5). **l–p** Femora from 23-weeks-old male Cre-control (Cre-Ctrl), *Gpr133/Adgrd1*^*tm1c*^ (+/+) and osteoblast-precursor-specific *Gpr133/Adgrd1*^*tm1d*^ KO (fl/fl) mice were examined by µCT. **l** Representative 3D reconstructions of the trabecular compartment and the femoral midshaft. (**m**) Ct. Th, (**n**) Tb.N, (**o**) BV/TV and (**p**) BMD were measured at the femoral midshaft. (*n* = 9 per group). **q** A three-point bending test was performed on femora as an indicator of bone strength and stiffness. Figure for experimental design was created with BioRender. (*n* = 9 per group). The maximum load (F_max_) tolerated by femora of (**r**) constitutive or (**s**) osteoblast-precursor-specific Het and Homo *Gpr133/Adgrd1* KO compared to WT controls were determined. (*n* = 9 per group). Data information: All data are presented as mean ± SD, each dot indicates an individual mouse. Statistical analysis was performed using one-way ANOVA: *p < 0.05; **p < 0.01; ***p < 0.001 comparing Het/ Homo vs. WT and fl/fl vs Cre-Ctrl or +/+

To investigate the molecular mechanisms underlying the influence of GPR133/ADGRD1 on human height and bone mineral density, we first established a constitutive receptor-deficient LacZ reporter mouse line (constitutive *Gpr133/Adgrd1* KO) to reflect the ubiquitous genetic deficiency in humans (supplementary Fig. 1a–c). LacZ staining in transgenic mice indicated *Gpr133/Adgrd1* expression in the ribs and vertebrae (Fig. 1a–d), as well as in the nucleus pulposus (Fig. 1a).

Heterozygous and homozygous *Gpr133/Adgrd1*-deficient mice displayed no obvious phenotypic differences compared to their wild-type (WT) littermates. A difference in body length was transiently observed in the first month after birth, but only in male animals (supplementary Fig. 1d), while limb length remained unaltered (supplementary Fig. 1e/f). Computed tomography scans revealed no changes in spinal axis in *Gpr133/Adgrd1*-deficient animals (supplementary Fig. 1g). To evaluate potential spine deformations, we quantified thoracocervical and thoracolumbar Cobb angles in sedated animals, which are formed by the most tilted vertebrae at the top and bottom of a spinal curvature. These also showed no statistically significant difference between WT and KO mice (supplementary Fig. 1h). Since neither spine deformation nor bone length in mice can be attributed to the length association reported in human cohorts, we assessed the nucleus pulposus morphology and resistance to pressure using atomic force microscopy as this structure is known to contribute to diurnal height changes in humans.^21^ Indeed, vacuolated nucleus pulposus cells showed reduced size and stiffness (supplementary Fig. 1i/j), which could account for the observed genome-wide association of SNPs in the *GPR133*/*ADGRD1* gene and body length in human populations but would not necessarily be detectable in a mouse model. As such, the role of GPR133/ADGRD1 in height changes and potential implications in vertebral disc pathologies will have to be studied in a different model system.

Besides an expression in vertebrae, we also detected *Gpr133/Adgrd1* transcripts in the tibia, femur, calvaria, and costal cartilage of 23-weeks-old WT mice (Fig. 1e), indicating a role for GPR133/ADGRD1 in bone homeostasis. Constitutive *Gpr133/Adgrd1* deletion resulted in decreased cortical bone thickness in 22-weeks-old male mice accompanied by reduced trabecular number as well as thickness, while trabecular separation was increased (Fig. 1f–h, supplementary Fig. 2i/j). The apparent loss of bone structure was further confirmed by a statistically significant reduction in bone volume (Fig. 1i) and significantly reduced bone mineral density (Fig. 1j) in KO animals.

To understand which cell population within the bone contributes to the observed phenotype we quantified *Gpr133/Adgrd1* transcript levels in different primary cell populations and representative cell lines. *Gpr133/Adgrd1* mRNA expression was detected in bone marrow mesenchymal stem cells (BM-MSCs), primary osteoblasts differentiated from BM-MSCs, undifferentiated and differentiated MC3T3 cells (osteoblast precursor cell line), primary bone marrow macrophages (BMMs), and primary osteoclasts. However, the osteocyte-like cell line MLO-Y4 and the osteoclast-like cell line RAW 264.7 only expressed trace levels of *Gpr133/Adgrd1* mRNA (Fig. 1k). Thus, we suspect the major functional impact on bone formation coming from the osteoblast cell population. We employed an osteoblast precursor-specific *Gpr133/Adgrd1* KO mouse line, which was based on the Cre-deleter Osx-Cre mouse line, to investigate this specific cellular contribution to the observed bone phenotype. To this end, constitutive *Gpr133/Adgrd1* KO mice were first crossed with Flp-mice, to remove the FRT-flanked selection cassette containing the SV40 polyadenylation sequence (supplementary Fig. 2a/b). This “conditional ready” *Gpr133/Adgrd1*^*tm1c*^ (+/+) mouse line was then crossed with the Cre-deleter Osx-Cre (Cre-Ctrl) to create the Osx-Cre;Gpr133/Adgrd1^tm1d^ KO (fl/fl) animals (see methods for more details, supplementary Fig. 2b–h). As the Cre-deleter Osx-Cre mouse line has been suggested to display altered tissue functions,^22^ we included animals from this genetic background (Cre-Ctrl) in our experiments to distinguish the effects of the genetic background from those of the *Gpr133/Adgrd1* KO phenotype. The male osteoblast precursor KO mice mirrored the phenotypes seen in microcomputed tomography (µCT) analysis of constitutive *Gpr133/Adgrd1* KO animals (Fig. 1l–p, supplementary Fig. 2k/l). Despite the seemingly moderate changes in the cortical structures (on average 4% reduction in cortical BMD and 10% reduction in cortical thickness), three-point bending tests of femurs from constitutive and osteoblast-specific heterozygous and homozygous *Gpr133/Adgrd1* KO mice revealed reduced cortical bone strength and elastic modulus (Fig. 1q–s, supplementary Fig. 2m/n), indicating a functional relevance of the cortical parameters. Collectively, these findings reflect an osteopenic phenotype, which was also observed in female mice (supplementary Fig. 3). In both sexes, heterozygous mice exhibited an intermediate phenotype. The osteopenic changes were also prominent in vertebral bone (supplementary Tables 1/2), indicating a global reduction in bone structure and strength.

### Reduced bone formation in *Gpr133/Adgrd1* KO mice is mainly driven by osteoblast dysfunction

**Fig. 2:**
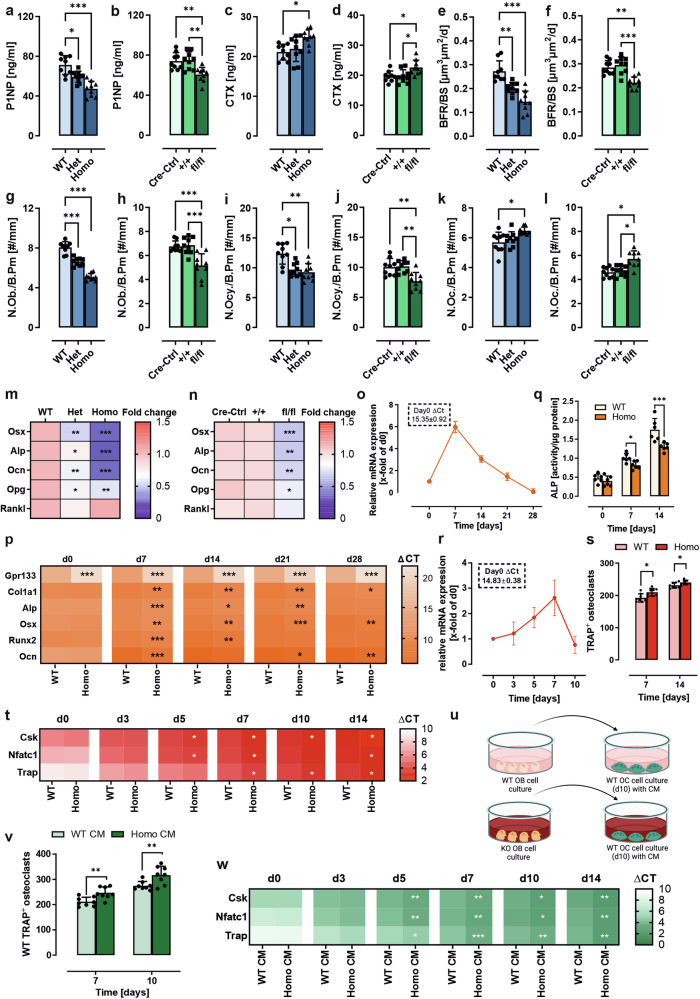
Bone depletion in constitutive and osteoblast precursor-specific *Gpr133/Adgrd1* knockout mice is mainly driven by osteoblast dysfunction. **a–n** 23-weeks-old male wild-type (WT), heterozygous (Het), or homozygous (Homo) *Gpr133/Adgrd1* knockout (KO) and Cre-control (Cre-Ctrl), *Gpr133/Adgrd1*^*tm1c*^ (+/+), and osteoblast-precursor-specific *Gpr133/Adgrd1*^*tm1d*^ KO (fl/fl) mice were examined. **a/b** Serum concentrations of the bone formation marker type 1 procollagen amino-terminal propeptide (P1NP) and (**c/d**) the bone resorption marker cross-linked C-telopeptide of type I collagen (CTX) were measured by ELISA. (**e/f**) Bone formation rate per bone surface (BFR/BS) assessed by calcein double labeling of tibial bone slides and compared between groups. Number of (**g/h**) osteoblasts per bone perimeter (N.Ob./B.pm), (**i/j**) osteocytes per bone perimeter (N.Ocy./B.pm), and (**k/l**) osteoclasts per bone perimeter (N.Oc./B.pm) were determined by Tartrate-resistant acid phosphatase (TRAP) staining of femoral bone slides. **m/n** Heat map of mRNA expression levels (fold change over WT/Cre-Ctrl mice) of osteoblast differentiation markers osterix (*Osx*), alkaline phosphatase (*Alp*), osteocalcin (*Ocn*), and the coupling factors osteoprotegerin (*Opg*) and receptor activator of NF-κB ligand (*Rankl*) in tibiae. (*n* = 9 per group). **o** *Gpr133/Adgrd1* mRNA expression during osteogenic differentiation of bone marrow-derived mesenchymal stromal cells was analyzed by qPCR. Results were calculated using the ΔΔCT method, normalized to β-actin mRNA, and presented as x-fold changes compared to d0. (*n* = 5). **p** Real-time PCR analysis was used to measure the mRNA expression of collagen type I alpha 1 (*Col1a1*), *Alp*, runt-related transcription factor 2 (*Runx2*), *Osx*, and *Ocn* in primary murine osteoblasts at days 0, 7, 14, 21, and 28 of differentiation. Results were calculated using the ΔCT method and normalized to β-actin mRNA. Data are presented as ΔCT values. (*n* = 6 per group). **q** ALP activity was assessed at days 0, 7, and 14 of differentiation. (*n* = 6). **r** *Gpr133/Adgrd1* mRNA expression during osteoclast (OC) differentiation in primary OCs was analyzed by qPCR. Results were calculated using the ΔΔCT method, normalized to β-actin mRNA, and presented as x-fold changes compared to d0. (*n* = 6). **s** TRAP positive multinucleated cells were identified as osteoclasts and counted. (*n* = 8). **t** Real-time PCR analysis was used to measure the mRNA expression of C-terminal Src family kinase (*Csk*), nuclear factor-activated T cells c1 (*Nfatc1*) and Tartrate-resistant acid phosphatase (*Trap*) in primary mouse osteoclasts at days 0, 3, 5, 7, 10 and 14 of differentiation. Results were calculated using the ΔCT method and normalized to β-actin mRNA. Data are presented as ΔCT values. (*n* = 8). **u** WT bone marrow-derived macrophages were cultured and differentiated into OC in the presence of WT or homozygous *Gpr133/Adgrd1* KO-derived conditioned medium (CM) of 7 days differentiated osteoblasts (OBs). Figure for experimental design was created with BioRender. **v** TRAP positive multinucleated cells were identified as osteoclasts and counted. (*n* = 8). **w** Real-time PCR analysis was used to measure the mRNA expression of *Csk*, *Nfatc1* and *Trap* in primary murine osteoclasts at days 0, 3, 5, 7, and 10 of differentiation. Results were calculated using the ΔCT method and normalized to β-actin mRNA. Data are presented as ΔCT values. (*n* = 8). Data information: All data are presented as the mean ± SD. Each dot represents an individual mouse. Statistical analysis was performed using (**a**–**n**, **q**, **u**) one-way ANOVA: *p < 0.05; **p < 0.01; ***p < 0.001 Het/Homo KO vs. WT control or fl/fl vs. Cre-Ctrl and +/+ and (**p**, **t**, **v**, **w**) two-way ANOVA: *p < 0.05; **p < 0.01; ***p < 0.001 WT vs. Homo KO or WT CM vs. Homo KO CM

Constitutive and osteoblast-specific deletion of *Gpr133/Adgrd1* resulted in lower plasma levels of the bone formation marker serum procollagen type I amino-terminal-propeptide (P1NP) and higher levels of the bone resorption marker CTX in male and female mice, indicating increased bone depletion (Fig. 2a–d, supplementary Fig. 4a–d). Dynamic bone remodeling was assessed using fluorescent calcein double-labeling in constitutive and osteoblast-specific KO mice, which showed statistically significant decreases in the bone formation rate-to-bone surface ratio (BFR/BS) (male: Fig. 2e/f, female: supplementary Fig. 4e–h), the mineralizing surface-to-bone surface ratio (MS/BS) and the mineral apposition rate (MAR) (supplementary Table 3) in male and female animals. Similar results were observed in the vertebrae of both sexes (supplementary Table 4).

In line with the observed expression of *Gpr133/Adgrd1* in osteoblasts, osteoclasts and differentiated MC3T3 cells (see Fig. 1k), significantly altered numbers of osteoblasts, osteoclasts and osteocytes were detected in tibiae and vertebrae of constitutive and osteoblast-specific KO mice of both sexes when compared to WT (male: Fig. 2g–l, female: Supplementary Fig. 4i–n, supplementary Table 4). The decreased mRNA transcript levels of the marker *Osx*, *Alp*, and *Ocn* in the bone tissue of either *Gpr133/Adgrd1* deficient mouse line in both sexes (male: Fig. 2m/n, female: supplementary Fig. 4o/p) are likely caused by the significantly reduced osteoblast number and function. Similarly, the significantly reduced transcript levels of osteoprotegerin (*Opg*) can be explained by the reduced osteoblast number or function and may contribute to the observed increase in osteoclasts in constitutive and osteoblast-specific KO mice, despite *Rankl* mRNA levels not being significantly altered (male: Fig. 2m/n, female: supplementary Fig. 4o/p).

To study the contribution of GPR133/ADGRD1 to osteoblast function we established an in vitro read-out based on primary BM-MSCs from WT and homozygous KO animals as well as MC3T3-E1 *Gpr133/Adgrd1*- and control siRNA-mediated knockdown (KD) cells (supplementary Fig. 4r). Osteogenic differentiation of MC3T3-E1 cells was induced by adding an osteogenic cocktail to the growth medium (see method section). *Gpr133/Adgrd1* transcripts are dynamically expressed during the course of osteoblast differentiation. BM-MSCs (Fig. 2o) and MC3T3-E1 cells (supplementary Fig. 4q) showed increasing *Gpr133/Adgrd1* mRNA levels, peaking at day (d) 7 of differentiation before returning to baseline. Similarly, in *Gpr133/Adgrd1* KO and KD cells, the expression levels of typical osteogenic markers (*Col1a1, Alp, Osx, Runx2, Ocn*)^2^ were consistently downregulated at d7 of differentiation compared to those from WT BM-MSCs, with this trend often persisting until full differentiation (Fig. 2p, supplementary Fig. 4s). In line with reduced *Alp* expression, the activity of this bone mineralization-supporting enzyme was significantly lower at both d7 and d14 (Fig. 2q, supplementary Fig. 4t) in *Gpr133/Adgrd1* KO and KD cells, which likely accounts for the observed decrease in collagen deposition (supplementary Fig. 4u/v) and impaired mineralization (supplementary Fig. 4w/x) as indicated by Sirius red and Alizarin red staining, respectively.

Because we also detected *Gpr133/Adgrd1* transcripts in primary osteoclasts (Fig. 1k), we followed the course of its expression during osteoclastogenesis. Expression of receptor transcripts in osteoclasts follows a similar pattern to that in osteoblasts but the maximum increase seen at d7 is only half as high as in osteoblasts (Fig. 2r, compare Fig. 2o). *Gpr133/Adgrd1* deficiency resulted in an increased number of TRAP-positive osteoclasts at d7 and d14 of the differentiation protocol (Fig. 2s), indicating faster or more efficient maturation. In line with this, expression of differentiation markers such as C-terminal Src family kinase (*Csk*), nuclear factor-activated T cells c1 (*Nfatc1*) and Tartrate-resistant acid phosphatase (*Trap*) in osteoclasts from homozygous KO mice displayed a significant increase with peak upregulation coinciding with the peak of *Gpr133/Adgrd1* expression (Fig. 2t). However, the differences in gene expression and osteoclast maturation between WT and KO cells were smaller than those observed in osteoblasts.

In bone osteoclast activation is highly regulated by soluble proteins from maturing osteoblasts.^2^ Among others, secreted RANKL binds to osteoclast precursor-expressed RANK to induce osteoclast differentiation, while OPG from osteoblasts masks RANKL to block this effect. We observed in our constitutive and osteoblast-specific KO mouse lines a significant reduction in OPG while RANKL remained mostly unchanged (see Fig. 2m/n), which can explain the induction of osteoclast differentiation and activity observed after incubation with conditioned media from WT and KO primary osteoblast cultures (Fig. 2u-w).

### *GPR133/Adgrd1* governs osteoblast differentiation and function through a cAMP-dependent modulation of the ß-catenin pathway

**Fig. 3:**
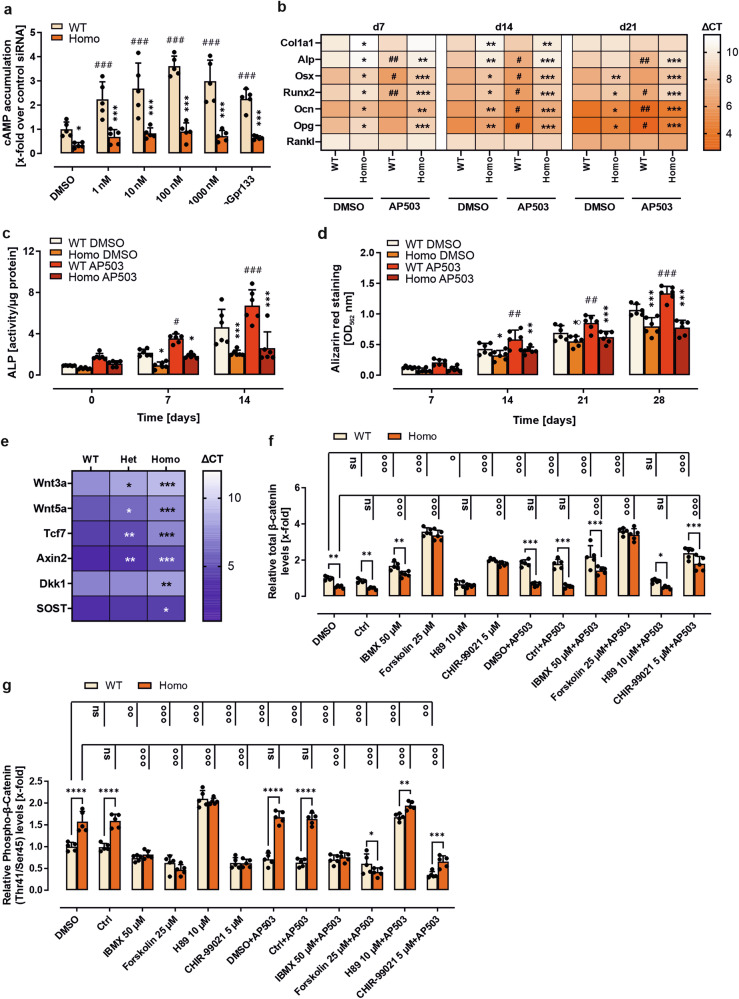
Osteoblast function is governed by GPR133/ADGRD1-mediated increase in cAMP-dependent ß-catenin signaling. **a** cAMP accumulation in 7-day differentiated primary bone marrow mesenchymal stem cells (BM-MSCs) derived from wild-type (WT) and homozygous (Homo) *Gpr133/Adgrd1* knockout (KO) mice. Cells were treated with different concentrations of AP503 or 1 mM *Stachel*-derived peptide pGPR133. (*n* = 5). **b** Real-time PCR analysis of mRNA expression levels of collagen type I alpha 1 (*Col1a1*), alkaline phosphatase (*Alp*), runt-related transcription factor 2 (*Runx2*), osterix (*Osx*), osteocalcin (*Ocn*), osteoprotegerin (*Opg*), and receptor activator of NF-κB ligand (*Rankl*) in BM-MSCs derived from WT and Homo *Gpr133/Adgrd1* KO mice at days 7, 14, and 21 of differentiation. Results were calculated using the ΔCT method, normalized to β-actin mRNA. (*n* = 6). **c** ALP activity was assessed at days 0, 7, and 14 of differentiation. (*n* = 6). **d** Mineralization capacity of osteoblasts was determined by Alizarin red staining at days 7, 14, 21, and 28 of differentiation. (*n* = 6). **e** The mRNA expression of Wnt signaling genes *Wnt3a*, *Wnt5a*, transcription factor 7 (*Tcf7*), *Axin2*, and Wnt inhibitors Dickkopf-1 (*Dkk1*) and sclerostin (*Sost*) in tibial bone tissue obtained from 23-weeks-old male WT, Het, or Homo *Gpr133/Adgrd1* KO mice was quantified by real-time PCR. Data are represented as the mean ± SD of ΔCT, normalized to β-actin mRNA. (*n* = 6). (**f/g**) Relative total and phosphorylated (Thr41/Ser45) β-catenin protein levels of 7-day differentiated primary BM-MSCs from WT and homozygous *Gpr133/Adgrd1* KO mice were determined by a cell-based ELISA. Cells were stimulated with AP503 (1 µM) and signaling pathways were probed using phosphodiesterase inhibitor IBMX, adenylyl cyclase activator forskolin, protein kinase A inhibitor H89 or Glycogen synthase kinase-3 inhibitor CHIR-99021 all at given concentrations. (n = 5). Data information: Data are presented as the mean ± SD, performed in triplicate per group. Statistical analysis was performed using two-way ANOVA: *p < 0.05; **p < 0.01; ***p < 0.001 Homo/Het KO vs. WT, and ^#^/°*p* < 0.05; ^##^/°°*p* < 0.01; ^###^/°°°*p* < 0.001 basal vs stimulated. n.s. no significance

Since GPR133/ADGRD1 also regulates cAMP levels in osteoblasts (supplementary Fig. 5a/b), we utilized our recently discovered G_s_ protein-biased agonist, AP503,^20^ to investigate the effects of direct receptor activation on cAMP levels in osteoblasts. This small molecule was identified through a structure-based virtual screening approach and validated for G-protein selectivity and potential cross-reactivity with other adhesion GPCRs. In WT to osteoblasts differentiated BM-MSCs and MC3T3 cells, AP503 induced significantly increased cAMP accumulation levels with 1 nM and 100 nM, respectively. In contrast, a concentration of 1 mM was needed to reach comparable cAMP levels with the agonistic *Stachel*-derived peptide pGPR133 (Fig. 3a, supplementary Fig. 5e). No detectable response was observed in *Gpr133/Adgrd1*-deficient cells for either agonist, despite similar cell numbers (supplementary Fig. 5c/d).

In addition, AP503 enhanced the differentiation of WT primary osteoblasts and MC3T3 cells, as evidenced by a significant upregulation of differentiation marker gene expression (Fig. 3b, supplementary Fig. 5f), increased ALP activity (Fig. 3c, supplementary Fig. 5g), and enhanced mineralization, as shown by Alizarin Red staining (Fig. 3d, supplementary Fig. 5h).

Since GPCR-mediated signals involved in osteoblast differentiation, bone development, homeostasis, and remodeling are typically attributed to the Wingless, Int-1 (Wnt) pathway,^23^ rather than to cAMP-mediated mechanisms, we examined selected components of Wnt signaling (*Wnt3a, Wnt5a, transcription factor (TCF)7, Axin2*) as well as its inhibitors (*dickkopf* (*Dkk)1, sclerostin (Sost)*) in osteoblasts from homozygous *Gpr133/Adgrd1* KO mice and compared them to WT conditions. All were significantly downregulated (Fig. 3e). Although cAMP and ß-catenin are often regarded as opposing signals in cell differentiation, cross-talk between the pathways has been documented, with cAMP-dependent protein kinase A (PKA) phosphorylating ß-catenin to prevent its degradation.^24^

To further explore this interaction downstream of GPR133/ADGRD1 in differentiated primary osteoblasts, we employed a PKA inhibitor (H89) and agents that elevate cAMP independently of receptor signaling (IBMX, forskolin). We also applied a glycogen synthase kinase 3 (GSK3) inhibitor (CHIR-99021) to prevent ß-catenin degradation. We then compared basal and AP503-stimulated ß-catenin levels of WT and KO primary osteoblasts under these conditions (Fig. 3f).

Basal ß-catenin levels were significantly reduced in KO cells and could not be increased by AP503 stimulation, unlike in WT cells. Both IBMX and forskolin significantly elevated ß-catenin levels in WT and KO cells, though only forskolin abolished the difference between *Gpr133/Adgrd1*-deficient and WT cells. Similarly, GSK3 inhibition rescued ß-catenin levels in KO cells, while PKA inhibition reduced ß-catenin levels in WT cells and blocked AP503-mediated increases.

Changes in total ß-catenin levels correlated with phosphorylation at Thr^41^ and Ser^45^ (Fig. 3g), which promotes degradation. As expected, GSK3 inhibition significantly decreased ß-catenin phosphorylation in both KO and WT cells. In contrast, PKA inhibition led to significantly increased phosphorylation of Thr^41^/Ser^45^. Additionally, cAMP-elevating agents (IBMX, forskolin) reduced ß-catenin phosphorylation in both cell types, while AP503 reduced phosphorylation only in WT cells. In sum, our findings support a mechanism in which GPR133/ADGRD1 stimulation leads to cAMP-mediated PKA activation and interferes with GSK3-dependent phosphorylation of ß-catenin at Thr^41^/Ser^45^. This blocks its degradation, resulting in higher levels of total ß-catenin.

### Activation of GPR133/ADGRD1 in osteoblasts is induced by its interaction partner PTK7 and mechanical force

In glioblastoma GPR133/ADGRD1 can be activated by its extracellular binding partner, the transmembrane protein PTK7.^18^ In combination with external forces, the interaction of PTK7 with GPR133/ADGRD1 in *trans* likely releases the N-terminal fragment, thereby exposing the internal agonistic *Stachel* sequence. Similar to *Gpr133/Adgrd1*, *Ptk7* is expressed in the femur, tibia, and calvaria of 12–16-weeks-old WT mice (supplementary Fig. 6a). Both BM-MSCs and MC3T3 cells show dynamic *Ptk7* expression during osteogenic differentiation with considerable overlap to *Gpr133/Adgrd1* transcripts (supplementary Fig. 6b/c; Fig. 2o and supplementary Fig. 4q). Knockdown of either the receptor or its ligand in MC3T3 cells led to a comparable reduction in osteogenic markers, including *Col1a1, Alp, Osx, Runx2*, and *Ocn* (supplementary Fig. 6d/e; supplementary Fig. 4s). Functional parameters, such as ALP activity, collagen deposition, and mineralization capacity, were also significantly reduced following *Ptk7* KD, compared to control-transfected cells (supplementary Fig. 6f-h).

To determine whether this phenotypic overlap is mediated by direct interaction between these membrane proteins, we first used transgenic knock-in mice expressing a GFP-tagged version of GPR133/ADGRD1 (GPR133^GFP^ mice^20^) and assessed co-expression of GPR133/ADGRD1 and PTK7 in situ. In bone tissue, we detected both *cis* and *trans* expression patterns for these molecules (supplementary Fig. 6i/j).

**Fig. 4:**
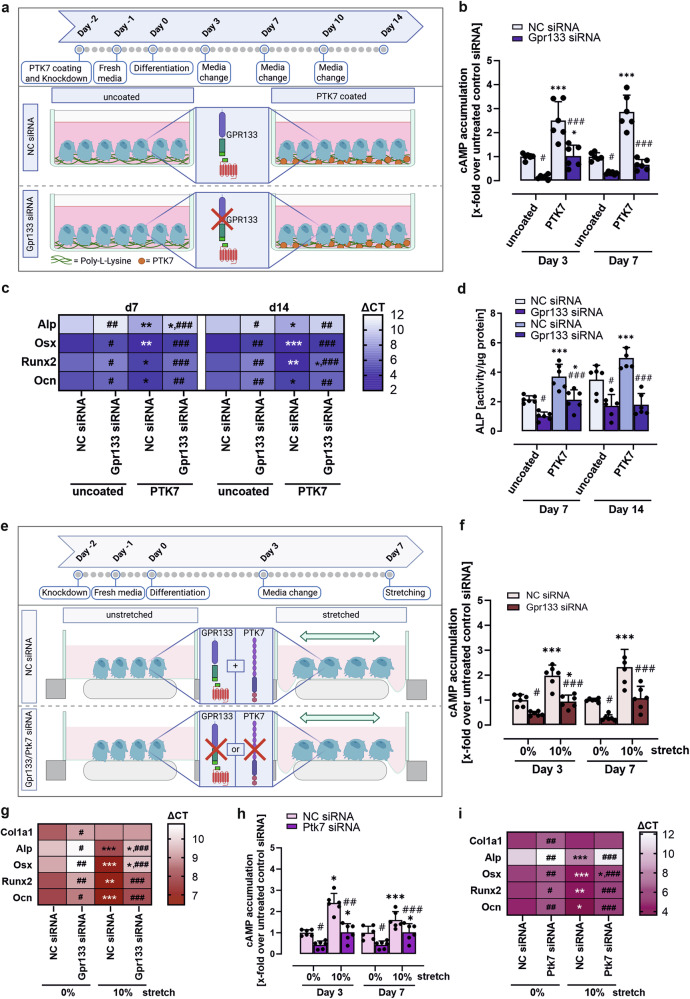
PTK7 and mechanical strain are important for *Gpr133/Adgrd1*-mediated osteoblast differentiation. **a** Experimental design for PTK7 coating and *Gpr133/Adgrd1* knockdown (KD) in MC3T3 cells (referring to **b**–**d**). Figure created with BioRender. **b** cAMP accumulation in 3- and 7-day differentiated negative control- (NC) and *Gpr133/Adgrd1* siRNA-transfected MC3T3 cells. (*n* = 6 per group). **c** Real-time PCR analysis of mRNA expression levels of collagen type I alpha 1 (*Col1a1*), alkaline phosphatase (*Alp*), runt-related transcription factor 2 (*Runx2*), osterix (*Osx*), and osteocalcin (*Ocn*) in NC- and *Gpr133/Adgrd1* siRNA-transfected MC3T3 cells at days 7 and 14 of differentiation. Results were calculated using the ΔCT method, normalized to β-actin mRNA. (*n* = 6 per group). **d** ALP activity was assessed at days 7 and 14 of differentiation in NC- and *Gpr133/Adgrd1* siRNA-transfected MC3T3 cells. (*n* = 6 per group). **e** Experimental design for mechanical strain and *Gpr133/Adgrd1* KD in MC3T3 cells (referring to **f**–**i**). Figure created with BioRender. **f** cAMP accumulation in 3- and 7-day differentiated NC- and *Gpr133/Adgrd1* siRNA-transfected MC3T3 cells subjected to 8 h of 10% or 0% cyclic tensile strain. (*n* = 6 per group). **g** mRNA expression levels of *Col1a1, Alp, Runx2, Osx*, and *Ocn* were measured in NC- and *Gpr133/Adgrd1* siRNA-transfected MC3T3 cells subjected to 8 h of 10% or 0% cyclic tensile strain at day 7 of differentiation. Results were calculated using the ΔCT method, normalized to β-actin mRNA. (*n* = 6 per group). **h** cAMP accumulation in 3- and 7-day differentiated NC- and *Ptk7* siRNA-transfected MC3T3 cells subjected to 8 h of 10% or 0% cyclic tensile strain. (*n* = 6 per group). **i** mRNA expression levels of *Col1a1, Alp, Runx2, Osx*, and *Ocn* were measured in NC- and *Ptk7* siRNA-transfected MC3T3 cells subjected to 8 h of 10% or 0% cyclic tensile strain at day 7 of differentiation. Results were calculated using the ΔCT method, normalized to β-actin mRNA. (*n* = 6 per group). Data information: All data are presented as mean ± SD, performed in triplicate per group. Statistical analysis was performed using two-way ANOVA: *p < 0.05; **p < 0.01; ***p < 0.001 basal vs stimulated (PTK7 coating or stretch), and ^#^*p* < 0.05; ^##^*p* < 0.01; ^###^*p* < 0.001 NC-siRNA vs. KD. n.s. no significance

For functional evaluation, we cultured control-transfected and *Gpr133/Adgrd1* KD MC3T3 cells on PTK7-coated plates (Fig. 4a). In control cells (NC siRNA), cAMP accumulation significantly increased after 3 and 7 days of differentiation on PTK7-coated plates. This response was markedly diminished in *Gpr133/Adgrd1* KD cells at d3 and undetectable at d7 (Fig. 4b). Moreover, PTK7 coating enhanced the expression of *Col1a1, Alp, Osx, Runx2*, and *Ocn* on d7 and d14, accompanied by elevated ALP activity (Fig. 4c/d). These positive effects on osteoblast differentiation and function were largely lost in the absence of *Gpr133/Adgrd1*, suggesting that the GPR133/ADGRD1–PTK7 interaction is critical for osteoblast differentiation and function.

Mechanical strain has previously been shown to promote MSC differentiation into osteoblasts (reviewed in ref.^25^). Given that GPR133/ADGRD1 has been reported to increase cAMP levels in response to mechanical forces,^16,17^ we next investigated whether GPR133/ADGRD1 mediates mechanoresponses in MC3T3 cells and whether *Gpr133/Adgrd1* KD alters differentiation under mechanical stimulation compared to controls. As the magnitude of cyclic tension force differentially affects OB differentiation,^26,27^ we first applied varying degrees of cyclic strain (0.1 Hz at 5%, 10%, or 15% elongation) on d7 for 8 h using the Flexcell® Tension System. Both 5% and 10% elongation significantly increased the expression of *Alp, Runx2, Osx*, and *Ocn*, with the strongest effect at 10% elongation (supplementary Fig. 6k). In contrast, 15% elongation impaired OB differentiation. Mechanical strain also significantly increased *Gpr133/Adgrd1* and *Ptk7* mRNA expression at 5% and 10% elongation (supplementary Fig. 6l/m).

Next, we applied mechanical strain to control-transfected, *Gpr133/Adgrd1* KD, and *Ptk7* KD MC3T3 cells (Fig. 4e). In control cells, mechanical strain significantly elevated cAMP levels on d3 and d7, whereas *Gpr133/Adgrd1*-deficient cells displayed a significantly lower response (Fig. 4f). Furthermore, *Gpr133/Adgrd1* KD abolished the differentiation-promoting effects of mechanical strain in MC3T3 cells (Fig. 4g). Similar impairments were observed in *Ptk7*-deficient cells (Fig. 4h/i), further supporting the concept of functional interplay between GPR133/ADGRD1 and PTK7 in mechanotransduction. Of note, the residual cAMP induction in siRNA transfected cells can be explained by the remaining transcripts in KD cells, as differentiated primary BM-MSCs derived from homozygous *Gpr133/Adgrd1* KO mice showed no response to mechanical strain, while WT BM-MSCs displayed the expected increase (supplementary Fig. 6n). As KD of *Ptk7* is even less efficient than *Gpr133/Adgrd1* KD and both of them decrease over time of differentiation (see supplementary Fig. 4r/s and supplementary Fig. 6d/e for *Gpr133/Adgrd1* and *PTK7*, respectively) we suspect that the same explanation holds true for observed residual activity in *Ptk7* KD cells.

### AP503 activates GPR133/ADGRD1 in vivo to enhance bone formation and counteract osteoporosis

**Fig. 5:**
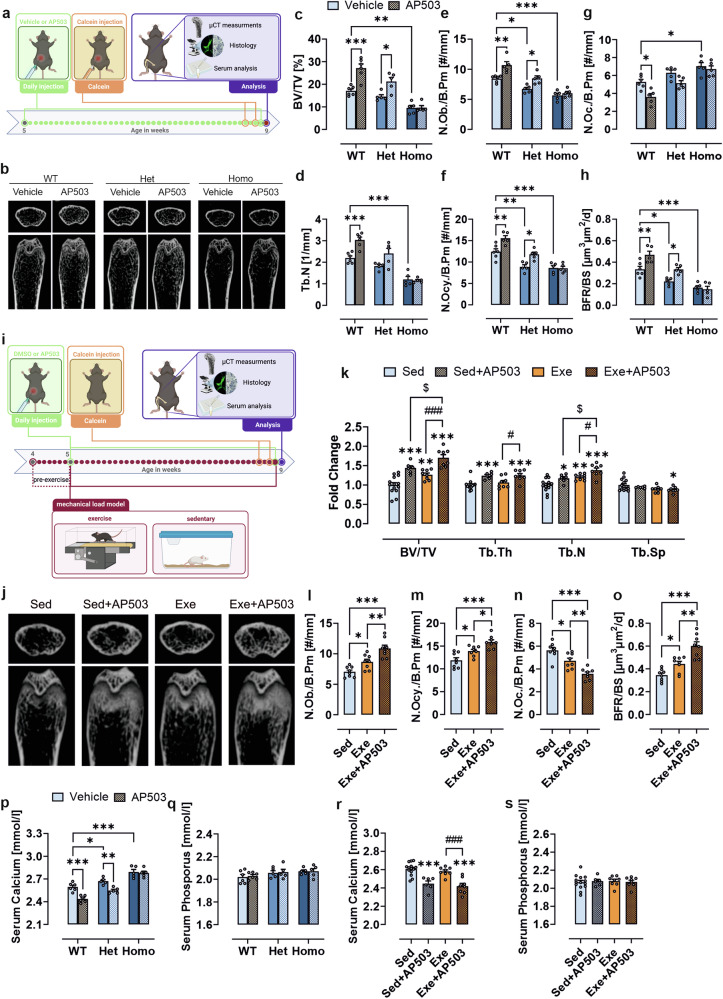
AP503 increases bone mass in male mice via GPR133/ADGRD1. **a** Experimental scheme for in vivo treatment (referring to data in b-h). Male wild-type (WT), heterozygous (Het), or homozygous (Homo) *Gpr133/Adgrd1* knockout (KO) mice were examined. 5-weeks-old WT, Het or Homo mice were intraperitoneally injected with vehicle or AP503 (2 mg/kg) every day for 4 weeks. Figure created with BioRender. **b–h** Femora from 9-weeks-old male mice were examined. **b** Representative µCT images of distal femurs (cross sections and longitudinal sections). **c** Bone volume/total volume (BV/TV) and (**d**) trabecular number (Tb.N.) were assessed in the distal femur using µCT. **e** Number of osteoblasts per bone perimeter (N.Ob./B.pm), (**f**) number of osteocytes per bone perimeter (N.Ocy./B.pm), and (**g**) number of osteoclasts per bone perimeter (N.Oc./B.pm) were determined by Tartrate-resistant acid phosphatase (TRAP) staining of femoral bone slides. **h** Bone formation rate per bone surface (BFR/BS) was assessed by calcein double labeling of tibial bone slides and compared between groups. **i** Experimental scheme for mechanical load model (referring to data in j-o). 4-weeks-old WT male mice were divided into 4 groups: sedentary control (Sed), Sed+AP503, exercise (Exe) and Exe+AP503. Mice in the Exe and Exe+AP503 group underwent treadmill running following a 5-day-per-week acclimatization protocol at 9 a.m., beginning with a 1-week pre-exercise phase, followed by a 4-week formal exercise period. Mice in the Sed and Exe groups were intraperitoneally injected daily with an equal vehicle, while those in the Sed+AP503 and Exe+AP503 groups received AP503 (2 mg/kg) daily. Figure created with BioRender. **j–o** Femora from 9-weeks-old male mice were examined. **j** Representative µCT images of distal femurs (cross sections and longitudinal sections). **k** Fold change of BV/TV, Tb.N, Tb.Th, and Tb.Sp compared to the Sed group. **l** N.Ob./B.pm, (**m**), N.Ocy./B.pm (**n**), and N.Oc./B.pm were determined by TRAP staining of femoral bone slides. **o** BFR/BS was assessed by calcein double labeling of tibial bone slides and compared between groups. **p** Serum calcium and (**q**) phosphate concentrations from control and AP503 treated male WT, Het or Homo *Gpr133/Adgrd1* KO mice. **r** Serum calcium and (**s**) phosphate concentrations from male WT mice following exercise and/or AP503 treatment. Data information: The data are presented as the mean ± SEM values, *n* = 5-14 mice per group. Each dot represents an individual mouse. The data were analyzed via one-way ANOVA with Tukey’s test. *p < 0.05, **p < 0.01, ***p < 0.001, ^#^*p* < 0.05, ^###^*p* < 0.001, ^$^*p* < 0.05

We intraperitoneally injected 5-weeks-old WT, heterozygous, and homozygous KO mice with 2 mg/kg of AP503 daily for 4 weeks to investigate the impact of GPR133/ADGRD1 activation on bone morphology (Fig. 5a). µCT analysis (Fig. 5b) revealed a significant increase in bone volume as well as trabecular number and thickness in WT and heterozygous animals upon AP503 treatment (Fig. 5c/d, supplementary Fig. 7a). Trabecular separation was significantly altered only in WT mice (supplementary Fig. 7b). Activation of GPR133/ADGRD1 in WT and heterozygous mice further led to a significant increase in osteoblast and osteocyte numbers and a reduction in osteoclasts (Fig. 5e–g). Additionally, bone formation rates (Fig. 5h) were elevated, and three-point bending tests revealed higher resistance to mechanical stress (supplementary Fig. 7c/d). In contrast, KO animals did not respond to AP503, mirroring our in vitro findings. Female mice showed similar results (supplementary Fig. 8).

Our in vitro data suggested that GPR133/ADGRD1 is activated by mechanical forces. To explore whether mechanical loading synergizes with GPR133/ADGRD1-mediated bone formation, we applied an exercise model previously shown to improve bone growth (Fig. 5i).^28^ µCT analysis of treated WT male mice revealed that AP503 increased bone volume, trabecular thickness, and trabecular number, while exercise alone increased bone volume and trabecular number but not thickness. Trabecular spacing remained unchanged by either stimulus (Fig. 5j/k, supplementary Fig. 7e-h). Interestingly, the combination of AP503 and exercise positively affected all parameters, suggesting a synergistic effect. This synergy was also reflected in osteoblast, osteocyte, and osteoclast numbers, as well as in the bone formation rate (Fig. 5l–o) and in F_max_ and E_mod_ values from three-point bending tests (supplementary Fig. 7i/j).

Blood samples from both experimental models revealed significantly elevated calcium ion levels in heterozygous and homozygous KO mice, while phosphate levels remained unchanged (Fig. 5p–s). Notably, only AP503 treatment—not exercise—reduced serum calcium ion levels in WT and heterozygous animals (Fig. 5p/r). This finding suggests elevated bone resorption through osteoclast activation in *Gpr133/Adgrd1*-deficient animals, in line with the combined impact of GPR133/ADGRD1 loss on osteoblasts and osteoclasts (see Fig. 2). These results support the potential of GPR133/ADGRD1 as a therapeutic target for osteoporosis, which is primarily driven by increased bone resorption, with reduced bone formation occurring secondarily.^29^

**Fig. 6:**
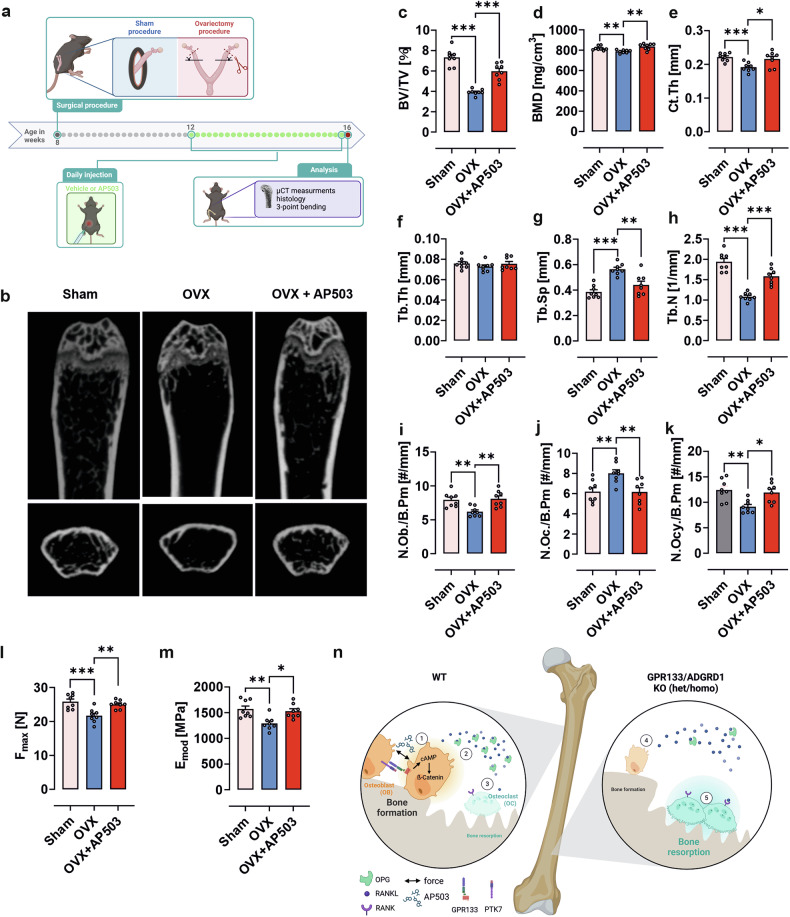
AP503 alleviates osteoporosis burden in vivo. **a** Overview of the experimental procedures for the ovariectomy mouse model. 8-weeks-old females were subjected to bilateral oophorectomy or sham operation, followed by 4 weeks of surgical recovery. After the recovery period, these mice with bilateral oophorectomy were intraperitoneally injected with vehicle or AP503 (2 mg/kg/day, i.p.) for 4 weeks before µCT test. Figure created with BioRender. **b-m** Femora from 16-weeks-old female mice were examined. **b** Representative µCT images of the trabecular compartment from 16-weeks-old sham, OVX and OVX + AP503 females. (**c**) Bone volume/total volume (BV/TV), (**d**) cortical bone mineral density (BMD), (**e**) cortical thickness (Ct.Th), (**f**) trabecular thickness (Tb.Th), (**g**) trabecular separation (Tb.Sp), and (**h**) trabecular number (Tb.N) of the femoral midshaft were assessed in the distal femur using µCT. **i** Number of osteoblasts per bone perimeter (N.Ob./B.pm), (**j**) number of osteoclasts per bone perimeter (N.Oc./B.pm), and (**k**) number of osteocytes per bone perimeter (N.Ocy./B.pm) were determined by Tartrate-resistant acid phosphatase (TRAP) staining of femoral bone slides. **l** The maximum load (Fmax) and (**m**) elastic modulus (Emod) were determined by 3-point bending test. **n** Summary of the proposed molecular mechanism through which GPR133/ADGRD1 controls bone formation. 1) Activation of GPR133/ADGRD1 through interaction with PTK7, mechanical forces or small molecule (AP503) induces osteoblasts differentiation through a cAMP-dependent ß-catenin pathway. 2) As a result osteoblasts secret an increased amount of osteoprotegerin (OPG), which masks receptor activator of nuclear factor-κB ligand (RANKL), thereby 3) reducing osteoclast (OC) activation. An additional suppression of OC activation is likely mediated through GPR133/ADGRD1-dependent cAMP-dependent inhibition of OC function. These combined effects mediate bone formation. 4) In the absence of a functional GPR133/ADGRD1 osteoblast differentiation and function is impaired, resulting among others in reduced expression of OPG, allowing now 5) for increased activation of OCs. Loss of GPR133/ADGRD1 in OCs leads to further activation, which taken together results in an osteopenic phenotype. Figure created with BioRender. Data information: The data are presented as the mean ± SEM values, *n* = 8 mice per group. Each dot indicates an individual mouse. The data were analyzed via one-way analysis of variance (ANOVA) with Tukey’s test. *p < 0.05, **p < 0.01, ***p < 0.001

As a proof of principle, we employed an ovariectomy mouse model, a standard experimental approach to mimic postmenopausal osteoporosis (Fig. 6a). As expected, WT mice subjected to ovariectomy at 8 weeks of age and treated with vehicle showed significantly reduced bone volume, bone mineral density, cortical thickness, and trabecular number, along with increased trabecular spacing, while trabecular thickness remained unchanged (Fig. 6b–h). Daily administration of AP503 for 4 weeks significantly alleviated all signs of osteoporosis, substantially restoring bone parameters toward WT levels. Likewise, ovariectomy-induced alterations in osteoblast, osteocyte, and osteoclast numbers (Fig. 6i–k) were completely reversed by AP503 treatment, resulting in a sham-operated-like resistance to three-point bending (Fig. 6l/m).

## Discussion

Osteoporosis results from an imbalance between bone formation and bone resorption. To maintain healthy bone, bone-forming osteoblasts and bone-resorbing osteoclasts must act in concert under the direction of osteocytes, which are embedded within the bone matrix (reviewed in ref. ^2^). Bone growth and formation are regulated by numerous hormones, growth factors, and cytokines, including insulin-like growth factor (IGF)1, bone morphogenetic proteins (BMPs), fibroblast growth factors (FGFs), Wnts, Notch, parathyroid hormone (PTH), as well as factors related to nutrition, mechanical loading, and aging. Here, we identify the G_s_-coupled adhesion GPCR GPR133/ADGRD1 as a novel regulator of osteoblast differentiation and function. The role of cAMP in osteoblast differentiation has yielded ambiguous results; some studies report an inhibition of mineralization in MC3T3 cells,^30^ while others find increased osteoblastogenesis.^31,32^ The timing of cAMP elevations likely accounts for these differences, as early stages of differentiation are negatively affected, while differentiated osteoblasts where reported to benefit from elevated cAMP levels.^33^ The GPCR-mediated signals known to promote osteoblast differentiation, bone development, homeostasis, and remodeling are usually attributed to the Wnt pathway.^23^ The PTH receptor also initiates G_s_ and Wnt signaling pathways,^34,35^ supporting resorption^36^ and formation.^37^ The frequency of PTH action on its receptor appears to be key in determining whether the action on bone is catabolic or anabolic; continuous administration has catabolic effects, while intermittent stimulation shows anabolic effects.^37,38^

We provide evidence that GPR133/ADGRD1 similarly couples cAMP-mediated β-catenin signaling, promoting osteoblastogenesis in primary osteoblasts (Fig. 3). Basal levels of both signaling molecules were markedly reduced in KO osteoblasts (Fig. 3), likely due to the overall impaired differentiation status of these cells and the absence of basal receptor activity. Based on our pathway analysis of β-catenin phosphorylation, we conclude that the reduction in β-catenin is primarily due to deficient upstream signaling rather than altered localization, as GSK-3 inhibition fully restored β-catenin levels in KO cells. Similarly, forskolin was able to normalize β-catenin levels, indicating that adenylyl cyclase expression is not dramatically affected by receptor KO. Nevertheless, reductions in other components of the cAMP and β-catenin pathways—including receptors and activators (Fig. 3e)—are also plausible. GPR133/ADGRD1 deficiency resulted in an osteopenic phenotype in both male (Fig. 1) and female mice (supplementary Fig. 3), accompanied by altered bone cellular composition (Fig. 2). We identified impaired osteoblast differentiation and function as the primary driver of osteopenia, as their dysfunction not only reduced bone formation but also significantly lowered *Opg* expression, likely contributing to increased osteoclast activation (Fig. 6n) as confirmed by co-culture experiments (Fig. 2). A direct role of GPR133/ADGRD1 in osteoclasts is also possible, as the receptor shows a similar, albeit less pronounced, expression pattern in osteoclasts, and KO of *Gpr133/Adgrd1* in these cells increased their activity and differentiation (Fig. 2), though to a lesser extent than in osteoblasts. Intermittent application of the novel GPR133/ADGRD1 agonist AP503 successfully rescued osteopenia in heterozygous mice and increased bone formation and strength in WT animals (Fig. 5). Furthermore, AP503 alleviated osteoporosis symptoms in an ovariectomy mouse model (Fig. 6). Unlike PTH receptor-mediated bone formation, GPR133/ADGRD1 activation integrates local signals such as mechanical forces and interaction with its endogenous ligand, PTK7^18^ (Fig. 4). A mouse exercise model suggests that mechanical forces and AP503 synergistically enhance bone formation (Fig. 5), indicating that each stimulus alone induces partial activation, whereas AP503 provides a more robust effect. While the structure of AP503 binding to the active state of GPR133/ADGRD1 has been resolved,^20^ it remains unclear what structural rearrangements occur upon mechanical stimulation, leaving the mechanism of this synergy unresolved. PTK7 is primarily known for its role in regulating planar cell polarity through non-canonical Wnt signaling,^39,40^ with known roles in cancer^41,42^ and embryonic development.^43,44^ To our knowledge, no prior reports have linked PTK7 to osteoblast differentiation and function. We show that PTK7 displays a dynamic expression pattern during osteoblast differentiation, overlapping with *Gpr133/Adgrd1* expression, and is essential for osteoblastogenesis (Fig. 4). We conclude that the interaction between GPR133/ADGRD1 and PTK7 allows for temporally controlled receptor activation during osteoblast differentiation.

At present, clinical translation of our findings on GPR133/ADGRD1-mediated bone formation remains a future goal. While mouse models are valuable for studying skeletal disorders and drug development, mouse bone structure differs slightly from humans, and interpretation of KO models carries limitations.^45^

Given that heterozygous KO mice exhibited intermediate phenotypes with several indicators of osteoporosis (Fig. 1), variants in *Gpr133/Adgrd1* could represent a genetic risk factor in humans. We identified several *Gpr133/Adgrd1* SNPs in healthy individuals that significantly reduce signaling.^12^ Such individuals may be predisposed to early-onset osteoporosis or heightened susceptibility to treatment-induced osteoporosis. For these populations, targeting GPR133/ADGRD1 may serve as a preventive strategy. Moreover, patients suffering from osteoporosis of various origins could benefit from this treatment, as we demonstrated that receptor activation reverses signs of postmenopausal osteoporosis in vivo. Additionally, this approach may improve muscle strength, as shown recently.^20^ Therefore, GPR133/ADGRD1 holds significant promise for geriatric medicine and for patients experiencing bone and muscle loss due to immobility or exposure to zero gravity. Future studies must focus on human patient specimen to validate the applicability of our results.

We conclude that the aGPCR GPR133/ADGRD1 substantially contributes to bone formation through local cell-cell interactions between osteoblasts and osteoclasts. The parallel receptor-mediated increase in cAMP levels in osteoblasts and osteoclasts resulting in cell-type-specific responses: increased activity in osteoblasts and suppression of osteoclast differentiation. This synergistic effect renders GPR133/ADGRD1 a promising candidate for treating osteopenic conditions.

## Materials and methods

### Experimental animals, ethics approval and study design

Mice were maintained under specific pathogen-free conditions with a 12 h light/dark cycle, at 21–24 °C, and 55% humidity with unrestricted access to food and water at Leipzig and Shandong University’s animals care facilities. All animal procedures were approved by the Animal Use Committee of Shandong University Cheeloo College of Medicine as well as the local authorities in the State of Saxony, Germany, in accordance with recommendations from the regional animal ethics committee (Landesdirektion Leipzig, TVV04/18, TVV43/18, TVV20/22, and TVV26/22) and the Government of the State of Saxony, Germany. Mouse sex is detailed in the figures or figure legends.

C57BL/6 N wild-type (WT) mice (Stock No.213) were purchased from Beijing Vital River Laboratory Animal Technology Co., Ltd. C57BL/6 J background Gpr133^-/-^ and Gpr133^GFP^ mice (with an N-terminal 3 × FLAG tag and a C-terminal GFP) were obtained from Cyagen (Suzhou, China). To eliminate potential strain-related effects, the mice were backcrossed with WT C57BL/6 J mice for over 12 generations. The C57BL/6 N embryonic stem cell line harboring the *Adgrd1tm1a(EUCOMM)Wtsi* allele was obtained from the European Conditional Mouse Mutagenesis Program (EUCOMM) and injected into 129/SV/J blastocysts. The *Adgrd1tm1a(EUCOMM)Wtsi* allele contains a cassette inserted after exon 7, which includes a mouse En2 acceptor splice site, a β-galactosidase reporter, a neomycin resistance gene, and a transcriptional termination SV40 polyadenylation sequence (supplementary Fig. 1a). The polyadenylation sequence prematurely terminates transcription, resulting in truncated transcripts that encode a nonfunctional reporter protein. This *knockout-first* allelic design is well-established and has been characterized in other genes.^46^ Since adhesion GPCRs in both mice and humans have complex genomic structures with many transcript variants caused by alternative splicing, exon skipping, and internal promoters,^47^ we characterized the transcripts generated in our genetic mouse model by RNA sequencing of urinary bladder transcripts to confirm the premature termination of transcription after exon 7, ensuring a functional knockout of *Gpr133/Adgrd1* (supplementary Fig. 1b). The promoter-driven cassette is flanked by FRT sites, while Exon 8 and the selection cassette are flanked by *lox*P sites (supplementary Fig. 1a). Genotyping PCR was performed with F 5’–CTTCAACACCACCAGCGATG-3’ and R 5’–GCACTGCATCTAGGTGGAGA-3’ to amplify a 361 bp- and a 351 bp-long fragment for WT and KO, respectively, with a *Sac*I restriction site only apparent in the KO allele. Digestion of the latter resulted in 286 bp- and 65 bp-long fragments (supplementary Fig. 1c).

To generate osteoblast precursor-specific *Gpr133/Adgrd1* KO mice, constitutive *Gpr133/Adgrd1* KO mice were first crossed with Flp mice to establish a “conditional ready” *Gpr133/Adgrd1*^*tm1c*^ (+/+) mouse line (supplementary Fig. 2a/b). This line was then crossed with the Cre-deleter Osx-Cre mouse strain^48^ to generate *Gpr133/Adgrd1*^*tm1d*^ KO (fl/fl) mice (supplementary Fig. 2c). Genotyping was carried out as follows: Using primer pairs Gpr133-neo-F (5’–CTGGATTCATCGACTGTGGC-3’) and Gpr133-ttR (5’– ACTTTGTACAAGAAAGCTGGG-3’) resulted in a 766-bp long band for the tm1a allele while Gpr133-F (5’–CTACTTTGTGGGTGGTGTCC-3’) and Gpr133-ttR (5’– GGGTGCTCACTCATCAATGG-3’) resulted in a 433-bp long band for tm1c (after FLP recombination) (supplementary Fig. 2d). Amplification with primers Gpr133-F and Gpr133-R resulted in a 536-bp long fragment tm1d (supplementary Fig. 2e). Once we verified the successful generation of *Gpr133/Adgrd1*^*tm1d*^ KO (fl/fl) mice we genotyped Osx-Cre using primers OsxCre-wildtypeF (5’–CCAGAGACGGAAATCCATCGCTCG-3’), OsxCre-mutantR (5’–CGGTCGATGCAACGAGTGATGAGG-3’) and OsxCre-wildtypeR (5’– CCTTCTCATTCCATGTCACCATGT-3’) which resulted in 225-bp long +/+-fragment and a 622-bp long mutant band in fl/fl and Cre-control mice, respectively (supplementary Fig. 2f). Proof of successful restoration of *Gpr133/Adgrd1* expression in *Gpr133/Adgrd1*^*tm1c*^ (+/+) mice is shown in supplementary Fig. 2g. Breeding pairs of the generated Gpr133fl;Osx:Cre mice were provided ad libitum access to drinking water containing 10 mg/mL doxycycline dissolved in 3% sucrose to suppress Cre activity during embryonic development. Additionally, Osx-Cre;Gpr133fl/fl offspring continued to receive doxycycline-treated water for up to five weeks post-weaning. Successful bone-specific deletion of *Gpr133/Adgrd1* in comparison to other receptor-expressing tissues was verified using qPCR (supplementary Fig. 2h).

### In vivo testing of AP503 impact on bone structure and composition

5-weeks-old male and female WT as well as constitutive heterozygous and homozygous KO mice were intraperitoneally injected with vehicle or AP503 (2 mg/kg, i.p.) every day for 4 weeks. Five and two days prior to euthanization, mice received intraperitoneal calcein injections (20 mg/kg body weight; Sigma-Aldrich, Munich, Germany).

### Exercise protocol

4-weeks-old male WT mice were divided into 4 groups: sedentary control (Sed), Sed+AP503, exercise (Exe) and Exe+AP503. Mice in the Exe and Exe+AP503 group underwent treadmill (Shenzhen Giant(Ju’An) Technology Co.,Ltd GAT-PB362) running following an acclimatization protocol of 5 sessions per week at 9 a.m., beginning with a 1-week pre-exercise phase, followed by a 4-week formal exercise period.^28^ Mice in the Sed and Exe groups were intraperitoneally injected daily with an equal volume of vehicle, while those in the Sed+AP503 and Exe+AP503 groups received 2 mg/kg AP503 daily.

Week 1 (Acclimation): 5-day progressive treadmill training. Weeks 2–5 (Formal Training): 5 sessions/week, 30 min/day at a constant speed of 16 m/min. Acclimation Phase (Week 1): Day 1 (15 min): First 5 min: Stationary on the treadmill. Subsequent 10 min: Speed starts at 6 m/min, increases by 2 m/min every 2 min, peaking at 12 m/min. Day 2 (15 min): Speed starts at 6 m/min for 2 min, increases by 2 m/min every 2 min, reaching 14 m/min. Day 3 (15 min): Speed starts at 6 m/min for 2 min, increases by 2 m/min every 2 min, reaching 16 m/min. Days 4–5 (30 min/day): Speed progression identical to Day 3 (6 → 16 m/min), but extended to 30 min. Post-Acclimation Training (Weeks 2–5) Frequency/Duration: 5 days/week, 30 min/session. Speed: Fixed at 16 m/min for all sessions. Equipment: Treadmill (model specified at first mention). Five and two days prior to euthanization, mice received intraperitoneal calcein injections (20 mg/kg body weight; Sigma-Aldrich, Munich, Germany).

### Ovariectomy mouse model and agonist testing

To establish cohorts of ovariectomized (OVX) females, bilateral ovariectomy was conducted at 8 weeks of age, followed by a 4-week postoperative recovery period. Concurrently, a group of females underwent sham surgery. After the recovery period, these mice with bilateral oophorectomy were intraperitoneally injected with vehicle or AP503 (2 mg/kg, i.p.) every day for 4 weeks before the µCT, histology and 3-point bending test.

### Analysis of Bone Morphometry and Biomechanical Properties

Femur and tibia lengths were measured using a caliper. To assess bone mass and microarchitecture, the distal femur and fourth lumbar vertebra (L4) were analyzed using µCT (vivaCT40, Scanco Medical, Brüttisellen, Switzerland) at 70 kVp X-ray energy, with an isotropic voxel size of 10.5 μm (114 mA, 200 ms integration time). For mice used in the ovariectomy mouse model and agonist testing, the distal femur was analyzed using microcomputed tomography (SKYSCAN 1276, Bruker micro-CT) at 65 kVp X-ray energy, with an isotropic voxel size of 20.7 μm (200 μA, 200 ms integration time). Trabecular and cortical bone parameters, such as bone volume/total volume (BV/TV), trabecular number (Tb.N), trabecular separation (Tb.Sp), trabecular thickness (Tb.Th), bone mineral density (BMD), and cortical thickness (Ct.Th), were quantified based on 100/50 scan slices, following standard Scanco Medical/Bruker protocols. For femoral analysis, trabecular parameters were measured in the metaphyseal region beginning 20 slices below the growth plate, while cortical parameters were assessed in the diaphyseal region, located midway between the femoral head and distal condyles. Trabecular bone of L4 was analyzed at the center of the vertebral body, encompassing 50 slices above and 50 slices below the midpoint.

To assess cortical bone strength, femurs underwent 3-point bending tests, while the fifth lumbar vertebra (L5) was used for compression tests (Zwick Roell, Ulm, Germany). Prior to biomechanical testing, femurs and the fifth lumbar vertebra (L5) were rehydrated overnight in PBS. The rehydrated femurs were positioned on two supports, with a distance of 6 mm between each of them. Vertebrae were positioned on the center of the lower plate. A vertical load was applied to the midshaft of the femur and to the L5 vertebral body via the superior end-plate, respectively. Once a preload of 1 N was achieved, measurements commenced and were performed at a loading rate of 0.05 mm/s until failure. Bone strength (F_max_) and stiffness (E_mod_) were calculated using TestXpert II software (version V3.7, Zwick Roell, Ulm, Germany).

### Bone histology and histomorphometry

Dynamic histomorphometric analysis was carried out as described in earlier studies.^49^ Mice were intraperitoneally injected with calcein (20 mg/kg body weight; Sigma-Aldrich, Munich, Germany) on days 5 and 2 prior to euthanasia. The tibiae and L3 vertebrae were fixed in 4% paraformaldehyde (PBS) for 48 h, followed by dehydration through a graded ethanol series. Bones were then embedded in methyl methacrylate (Technovit 9100, Heraeus Kulzer, Hanau, Germany) and sagittally sectioned at 7 μm to quantify calcein labeling in trabecular bone. The mineralized surface/bone surface (MS/BS), mineral apposition rate (MAR), and bone formation rate/bone surface (BFR/BS) were assessed 4 µm below the growth plate of the tibiae in a standardized area of trabecular bone (0.72 mm²) and from the center of the vertebrae (1.44 mm²), according to standardized fluorescence microscopy protocols (Keyence BZ-X800 microscope, Osaka, Japan).

In order to stain for tartrate-resistant acid phosphatase (TRAP), femurs and L4 vertebrae were fixed in 4% PBS-buffered paraformaldehyde, decalcified for 7 days using Osteosoft (Merck, Darmstadt, Germany), dehydrated through a graded ethanol series, and embedded in paraffin. In L4 vertebrae TRAP staining was applied to 2 μm-thick paraffin sagittal sections. For analysis, a region of 0.36 mm² beneath the growth plate and a 0.90 mm² area from the center of the vertebral body were evaluated using CellProfiler software (version 4.2.5). Terminology and quantification procedures followed the guidelines established by the Nomenclature Committee of the American Society for Bone and Mineral Research (ASBMR).^50^

### Immunofluorescence staining and confocal microscopy

Following sacrifice, the right femur was immersed in 4% paraformaldehyde for 48 h for fixation. The femur was then placed in EDTA decalcifying solution (Servicebio, G1105) for decalcification, with the solution being replaced every three days for a total of two weeks. The specimen was subsequently dehydrated in sucrose solutions with increasing concentrations (10%, 20%, and 30% sucrose in phosphate-buffered saline, PBS) for 24 h each and embedded in Tissue-Tek O.C.T. compound (Sakura Finetek USA, Torrance, CA) at the optimal temperature. Longitudinal sections (6 μm thick) of the proximal region of the specimen were prepared using a cryostat, collected on adhesion slides, and stored at *−*80 °C. Fluorescence immunostaining was conducted on the obtained sections. Femoral sections were blocked in PBS containing 10% goat serum and 0.4% triton X-100 for 30 min, then incubated overnight at 4 °C with primary antibodies: anti-GFP mouse antibody (Proteintech, 66002-1-Ig, 1:100) and anti-PTK7 Rabbit antibody (Proteintech, 17799-1-AP, 1:100) diluted in 2.5% BSA. After washing with PBS, sections were incubated for 2 h at 37 °C with secondary antibodies: goat anti-rabbit antibody (Invitrogen, 1:300) or goat anti-mouse antibody (Invitrogen, 1:300). Nuclei were counterstained with DAPI (1:2000), and fluorescence imaging was performed using an LSM980 laser scanning confocal microscope system (ZEISS, Germany). Image acquisition and analysis were conducted with ZEISS Zen software.

### Blood analysis

Mice were sedated with an intraperitoneal injection of ketamine (100 mg/kg; WDT eG, Garbsen, Germany) and xylazine (10 mg/kg; Rompun®, Bayer Vital GmbH, Leverkusen, Germany). Blood sampling was performed after euthanization via cardiac puncture. The collected blood was added to ice-cold EDTA (1.5 mg/ml blood) in 0.9% NaCl. The samples were centrifuged at 500 × *g* for 8 min, and the resulting plasma was transferred to fresh tubes and stored at *−*80 °C. Plasma concentrations of the bone turnover markers, procollagen type 1 amino-terminal propeptide (P1NP) and C-terminal telopeptide (CTX), were measured employing enzyme-linked immunosorbent assays (ELISAs) following the manufacturer’s instructions (IDS, Frankfurt/Main, Germany). To obtain serum calcium and phosphate content blood was centrifuged at 1500 x *g* to isolate serum, which was then preserved at *−*80 °C. The calcium (Beyotime, S1063S) and phosphorus (Beyotime, S0235S) contents were measured according to the corresponding instructions.

### Cultivation and functional testing of MC3T3 cells and primary mouse osteoblasts

The murine osteoblastic cell line MC3T3-E1 Subclone 4 was cultured in α-MEM medium supplemented with 2 mM L-glutamine, 10% fetal bovine serum (FBS), and 1.5% penicillin/streptomycin (Pen/Strep). Cells were maintained in a humidified atmosphere with 5% CO_2_ at 37 °C until they reached approximately 80% confluence. To induce osteogenic differentiation, an osteogenic cocktail was added to the growth medium. This cocktail contained 100 μM ascorbate phosphate, 10 mM β-glycerol phosphate (both from Sigma-Aldrich, Munich, Germany), and 5 ng/ml bone morphogenetic protein 2 (BMP2; PeproTech, Cranbury, USA). The medium was refreshed every other day.

Primary mesenchymal stromal cells were obtained by flushing the bone marrow from the hind legs of 12 to 16-weeks-old mice. Cells were cultured in growth medium (DMEM supplemented with 10% fetal bovine serum and 1.5% penicillin/streptomycin) at 37 °C in a humidified atmosphere containing 95% air and 5% CO_2_, until reaching approximately 80% confluence. For all experiments, 30,000 cells/cm² were seeded onto the different cell formats. After 24 h, osteoblast differentiation was induced by adding an osteogenic cocktail to the growth medium containing 100 μM ascorbate phosphate and 10 mM β-glycerol phosphate (both from Sigma-Aldrich, Munich, Germany). The medium was changed every other day.

Alkaline phosphatase (ALP) activity was assessed as a marker of osteogenic differentiation and cellular function. Primary osteoblasts were cultured under osteogenic conditions for 14 days. Following differentiation, cells were washed with phosphate-buffered saline (PBS) and lysed in ALP lysis buffer consisting of 10 mM Tris-HCl (pH 8.0), 1 mM MgCl_2_, and 0.5% Triton X-100. The lysates were homogenized by passage through a 24-gauge needle and centrifuged at 25,000 × g for 30 min at 4 °C. For the enzymatic reaction, 10 μL of the resulting supernatant was added to 90 μL of ALP substrate buffer containing 100 mM diethanolamine, 150 mM NaCl, 2 mM MgCl_2_, and 2.5 mg/mL p-nitrophenylphosphate. The reaction mixture was incubated at 37 °C for 30 min. ALP activity was determined by measuring the absorbance at 410 nm using a Tecan microplate reader (Tecan Group AG, Männedorf, Switzerland), reflecting the hydrolysis of p-nitrophenylphosphate. Results were normalized to the total protein content, which was quantified using the Pierce BCA Protein Assay Kit (Thermo Fisher Scientific, Schwerte, Germany) according to the manufacturer’s instructions.

Sirius Red staining was conducted to evaluate collagen secretion during osteogenic differentiation. Cells were differentiated for 7, 14, 21, and 28 days. At each time point, the culture medium was aspirated and cells were washed once with PBS. Subsequently, cells were stained with 0.1% Picro-Sirius Red dissolved in saturated aqueous picric acid for 1 h under gentle agitation using a microplate shaker. Following staining, excess dye was removed with 0.01 M HCl. To quantify collagen-bound dye, the stained material was solubilized in 200 µL of 0.1 M NaOH with shaking for 30 min. Absorbance was measured at 550 nm using a microplate reader.

To evaluate mineralization capacity, MC3T3-E1 cells or primary osteoblasts were cultured under osteogenic conditions for 7, 14, 21, or 28 days. At each time point, cells were fixed with 10% paraformaldehyde for 15 min at room temperature, followed by staining with 1% Alizarin red S solution (pH 5.5, Sigma-Aldrich, Munich, Germany) for 30 min. After staining, excess dye was removed by multiple washes with distilled water. To quantify mineral deposition, the bound Alizarin Red S was solubilized using cetylpyridinium chloride (Sigma-Aldrich, Munich, Germany), and absorbance was measured photometrically at 562 nm using a microplate reader.

### Osteoclast differentiation and TRAP staining

To generate osteoclasts, bone marrow cells were cultured in α-MEM supplemented with 10% FBS, 1% penicillin/streptomycin and 25 ng/ml macrophage colony-stimulating factor (MCSF, R&D Systems, Minneapolis, USA) for 48 h. Subsequently, cells were further treated with 50 ng/ml receptor activator of nuclear factor kappa-B ligand (RANKL, R&D Systems, Minneapolis, USA) for an additional 10 days to induce osteoclast differentiation. Tartrate-resistant acid phosphatase (TRAP) staining was performed on day 10 to identify osteoclasts. To this end, cells were fixed for 10 min at room temperature in acetone/citrate buffer containing 37% paraformaldehyde. Following two washes with tap water, TRAP staining was carried out for 20 min in the dark using the TRAP Staining Kit (Sigma-Aldrich, St. Louis, USA). Multinucleated ( ≥3 nuclei) TRAP-positive cells were identified and counted as osteoclasts. For indirect co-culture experiments, conditioned medium from 7-day-differentiated osteoblasts obtained from WT or homozygous *Gpr133* KO mice was collected and stored at *−*80 °C. To analyze the effect of osteoblast-derived factors on osteoclast differentiation primary osteoclasts were differentiated as described above with adding 50% conditioned medium with every media change.

### siRNA transfection

To suppress receptor mRNA expression, a small interfering RNA (siRNA)-mediated knockdown (KD) strategy was employed. Oligos of siRNA specifically targeting mouse *Gpr133/Adgrd1* and *Ptk7* were designed and provided by Origene (Rockville, USA), including scrambled control siRNA. Briefly, 10 nM of siRNA was transfected with RNAiMAX transfection reagent (Invitrogen, Waltham, MA, USA) into 100,000 MC3T3 cells/ml at 37 °C. After 24 h, an equal volume of fresh α-MEM medium was applied to the culture, and they were incubated for another 24 h. The next day (day 0), differentiation of MC3T3 cells or osteoblasts was induced using α-MEM supplemented with 100 μM ascorbate phosphate and 10 mM β-glycerol phosphate (both Sigma-Aldrich, Munich, Germany). Since the KD was no longer visible at day 14 of differentiation, we performed a second transfection on day 10 of differentiation. To do this, transfection was carried out using 10 nM of siRNA or the respective scrambled control. After 24 h, an equal volume of fresh α-MEM supplemented with 200 μM ascorbate phosphate and 20 mM β-glycerol phosphate was added to the culture.

### Stretch assays

Stretching experiments were performed with the FX-6000™ Tension System (Flexcell International, Hillsborough, NC). MC3T3-E1 cells and primary osteoblasts were seeded on collagen I-coated 6-well BioFlex culture plates with elastic bottoms (Flexcell International Corporation, Burlington, NC, USA) and differentiated for 3 or 7 days as described above. Cells were subjected to cyclic mechanical stretch at elongation magnitudes of 5%, 10%, and 15% using a frequency of 0.1 Hz (5 s stretch/5 s relaxation) for a total duration of 8 h. Control groups were seeded on identical plates and maintained under the same culture conditions without the application of mechanical stretch (0% elongation). Knockdown of *Gpr133/Adgrd1* or *Ptk7* was performed as described above, 48 h prior to differentiation (day 0).

### cAMP assay

MC3T3 cells (10,000 cells/well) were seeded and transfected in 96-well plates as described above. Briefly, 10,000 cells per well were transfected with either *Gpr133/Adgrd1* siRNA or a scrambled control. After 24 h, 100 µL of fresh α-MEM medium was added to the cells, and they were incubated for another 24 h. On the following day (designated as day 0), osteogenic differentiation was initiated as previously described, and cells were subsequently differentiated for 7 days. Primary cells were obtained and differentiated as described above.

To measure cAMP accumulation, MC3T3 or primary cells differentiated for 7 days were incubated in α-MEM or DMEM medium, respectively, including 1 mM 3-isobutyl-methyl-xanthine (IBMX) for 30 min. IBMX inhibits phosphodiesterases, thus blocking the degradation of cAMP. For peptide stimulation, the *Stachel*-sequence-derived peptide was diluted in IBMX-containing medium. The peptide solution from the purified powder was prepared by dissolving it in 100% DMSO, then diluting to 10 mM stock solutions in 50 mM Tris buffer (pH 8), with pH carefully controlled. The peptide concentration used in the assays was 1 mM, which contained 1% DMSO and 10% Tris buffer. Control conditions included 1% DMSO and 10% Tris buffer, without the peptide. For AP503 stimulation, different concentrations (1–1000 nM) dissolved in DMSO were used. Following stimulation, cells were lysed in LI buffer (PerkinElmer, Rodgau, Germany) and stored at −20 °C until further analysis. cAMP concentration was measured using the AlphaScreen cAMP assay kit (PerkinElmer, Rodgau, Germany) following the manufacturer’s instructions. The accumulated cAMP was quantified using a 384-well white OptiPlate microplate (PerkinElmer, Rodgau, Germany) and analyzed with the EnVision Multilabel Reader (PerkinElmer, Rodgau, Germany).

In experiments involving PTK7-coated culture vessels, well plates were first treated with a 0.01% solution of poly-L-lysine (PLL), then washed with distilled deionized water. Next, the plates were coated with 586 ng/cm² of PTK7 (R&D, Cat# 9799-TK-050). The plates were left to air-dry overnight and then rinsed once more with distilled deionized water before plating the cells.

### Total and phosphorylated β-catenin measurement

Total and phosphorylated β-catenin (phospho Thr^41^/Ser^45^) levels were analyzed using a commercially available Cell-Based ELISA Kit (Antibodies.com). Primary mesenchymal stromal cells (BM-MSCs) were obtained from 12 to 16-weeks-old mice and differentiated as described above. The primary cells were differentiated for 7 days. The following day, cells were stimulated for 5 h with a 1 mM peptide solution, 1000 nM AP503, or DMSO as control. Afterwards, the cell-based ELISA was carried out according to the manufacturer’s instructions, with primary antibodies incubated overnight at 4 °C. Measurements and data analysis were conducted as described in the protocol, including anti-GAPDH absorbance as an internal control and crystal violet staining for normalization. To explore the interactions downstream of GPR133/ADGRD1, primary cells were differentiated for 4 days and subsequently incubated for additional 3 days with 50 µM IBMX, 25 µM Forskolin, 10 µM PKA-Inhibitor H89 (Merck, Cat#B1427), 5 µM GSK3-inhibitor CHIR-99021 (Merck, Cat#SML1046), DMSO, 1000 nM AP503 or control.

### Transcript quantification through quantitative real-time PCR

Total RNA was extracted from cultured cells using the ReliaPrep RNA Miniprep Kit (Promega, Fitchburg, USA) following the manufacturer’s protocol. RNA concentration was quantified using a Nanodrop spectrophotometer (Peqlab, Radnor, USA). For RNA isolation from bone tissue, femurs and tibiae were flushed, and subsequently crushed in liquid nitrogen. The tissue remnants were then immersed in Trifast reagent (Peqlab, Radnor, USA), and RNA extraction was performed according to the manufacturer’s instructions. After ethanol precipitation, samples were further purified using the ReliaPrep RNA Miniprep Kit (Promega, Fitchburg, USA) following the manufacturer’s instructions. RNA (1000 ng) was then used for reverse transcription with the iScript cDNA Synthesis Kit (BioRad, Berkeley, USA) and subsequently analyzed by Luna Universal qPCR Master Mix-based real-time PCRs using the standard protocol (New England Biolabs, Ipswich, USA). Primer sequences are listed in supplementary Table 5. Quantitative real-time PCR was performed under the following thermal cycling conditions: an initial step of 5 min at 50 °C, followed by 2 min at 95 °C, and 45 cycles of 30 s at 95 °C and 30 s at 60 °C. A melting curve analysis was conducted with an initial step of 10 s at 90 °C, followed by 5 s at 60 °C, with a continuous temperature increase of 0.5 °C increments up to 95 °C. Gene expression levels were calculated using the comparative Ct (ΔΔCt) method. Results are presented as fold changes relative to β-actin, unless otherwise specified.

### Statistical analysis

Data are presented as means±standard deviation (SD) or standard error of the mean (SEM). Statistical analyses were performed using GraphPad Prism version 10.0 (GraphPad, La Jolla, CA, USA). A *p*-value of <0.05 was considered statistically significant. The specific test applied to each data set can be found in each figure legend.

## Supplementary information

Supplementary Materials

## Data Availability

All data and materials are available within the submitted materials or in the public repository Zenodo: 10.5281/zenodo.15607037. The RNA sequencing data is available under the GEO accession number: GSE298885.
