# Supplementary material for: The mechanosensitive adhesion G protein-coupled receptor 133 (GPR133/ADGRD1) enhances bone formation
Source: Signal Transduct Target Ther. 2025 Jun 30;10:199. doi: 10.1038/s41392-025-02291-y (PMC12206920; doi:10.1038/s41392-025-02291-y)
Supplement: Supplementary file 1 — Supplementary Materials [file 41392_2025_2291_MOESM1_ESM.docx]

Supplementary Materials for

The mechanosensitive adhesion G protein-coupled receptor 133 (GPR133/ADGRD1) enhances bone formation

Juliane Lehmann, Hui Lin, Zihao Zhang, Maren Wiermann, Albert M Ricken, Franziska Brinkmann, Jana Brendler, Christian Ullmann, Luisa Bayer, Sandra Berndt, Anja Penk, Nadine Winkler, Franz Wolfgang Hirsch, Thomas Fuhs, Josef Käs, Peng Xiao, Torsten Schöneberg, Martina Rauner, Jinpeng Sun, Ines Liebscher

Correspondence to: [ines.liebscher@medizin.uni-leipzig.de](mailto:ines.liebscher@medizin.uni-leipzig.de)

[sunjinpeng@sdu.edu.cn](mailto:sunjinpeng@sdu.edu.cn)

[juliane.lehmann@medizin.uni-leipzig.de](mailto:juliane.lehmann@medizin.uni-leipzig.de)

**This PDF file includes:**

Materials and Methods

Supplementary Text

Figures. S1 to S8

Tables S1 to S5

Materials and Methods

RNA-Seq analysis

For library preparation and sequencing, total RNA was extracted from mouse heart samples using the TRI Reagent according to the manufacturer’s instructions. The quality and quantity of the purified RNA were determined by measuring the absorbance at 260/280 nm (A260/A280) using a NanoDrop spectrophotometer. Indexed cDNA libraries with an average insert size of 300 bp were constructed using the TruSeq RNA sample preparation kits v2 (Illumina, San Diego, CA, USA). Libraries from 2-4-month-old female WT (n = 4) and KO (n = 4) mice were pooled and loaded onto two flow cell channels. A minimum of 7 million reads per library were sequenced.

After assigning reads to samples according to library index, we used a sequencing pipeline to trim the adapters and remove reads < 60 bp^1^. Reads were mapped to the mouse genome (July 2007 NCBI37/mm9) with Ensembl v66 annotations^2^ using TopHat 1.3.3^3^. We excluded reads that mapped to the mitochondrial genome and reads that did not map uniquely to the reference nuclear genome. The transcription level for each gene was obtained by intersecting mapping results with gene annotations using BEDTools^4^.

Body and bone length measurements

The development of body length and weight of newborn conscious mice was documented on different days, including days 5, 10, 15, 20, 30, 40, and 50 using a ruler (Herlitz, Pelikan Vertriebsgesellschaft mbH & Co. KG, Berlin, Germany) and an electronic scale (Sartorius CP4202S, Sartorius AG, Göttingen, Germany). The body length was determined by measuring the distance between the tip of the nose and the base of the tail while holding the mice by the scruff. For distinction, the extremities of all mice were tattooed on days 2 to 3 using a needle and ink. Litters with four or fewer mice were excluded from the measurement. The mice were genotyped between days 20 and 40. The lengths of the tibia and the femur of mice were measured with a caliper (Hilitand, China).

Computer tomography of mouse skeleton

Experiments were performed in a double-blinded setup. 7 male WT and homozygous KO mice aged 3 - 5 months were investigated. Raw data was obtained using a XR 4.0 expert unit (Phywe, Göttingen, Germany) equipped with a tungsten X-ray source using 35 kV and 1 mA. To reduce beam hardening, an additional 0.3 mm aluminum foil (Al 99.5, Reely/Conrad electronic SE, Hirschau, Germany) was used. The detector had an active range of 49.25 mm and a matrix of 1000 pixels. The SOD was 320 mm and the SDD 359 mm yielding a digital resolution of 43.9 µm per pixel. 450 projections were acquired (last angle 360°) with 1.4 s exposure time per projection. After sacrificing, each mouse was transferred into a home-built tube and then 3 scans were collected per specimen to sample the whole dimension from nose to tail with an overlapping area to reconstruct a final image. After reconstruction of the data with a BHC factor of 0.04, data were converted in HU scale, scans merged using custom-written Matlab (MathWorks, USA) scripts and ImageJ, and the mouse skeleton was then visually inspected in ImageJ and VolView (Kitware, USA).

Atomic/scanning force microscope (AFM) measurements

AFM measurements were done on a Nanowizard 4 (JPK, Germany) equipped with the 300x300x300 µm hybrid stage, mounted on a Zeiss Axio Zoom, V16 optical microscope. The samples were probed with CONT cantilevers (NanoWorld, CH) with a spring constant around 100 mN/m. The cantilevers were custom modified with a 6 µm polystyrene bead glued to the tip to increase the contact area. Force-indentation maps (100x100 µm) were recorded with 10 µm pixel spacing on an area of 100x500-700 µm stretching outwards from the center of the disc. Samples were freshly prepared for AFM imaging and glued with histoacryl (B. Braun, Germany) to a small piece of glass for immobilization during AFM measurement. Special care was taken to ensure the glue did not extend to the top surface of the disc. The mounted disc was then submerged in buffer, and a 5% CO_2_ atmosphere was created above the buffer during measurement. Nuclei were stained with Hoechst 33258 briefly before measurement, and the disc was imaged. Measurement time was kept below 90 minutes for all samples. After measurement, the disc was fixed overnight in formalin (HT5011, Sigma, Munich, Germany) for further imaging.

Force-indentation curves were analyzed with the JPK data processing software, and the Young’s modulus calculated by fitting the Hertz model to the data. Fitted data was further analyzed in a custom Matlab script (MathWorks, USA). The script sorted out curves where the cantilever failed to make contact, falsely reporting stiffness values below 1 Pa, and excluded areas with unphysiologically high stiffness (above 10 kPa). The edge between the nucleus pulposus and anulus fibrosus was clearly visible in the data by a marked increase in stiffness. The region of the map used for statistical analysis was manually selected to extend up to the point of this marked increase.

Confocal spinning disc microscope imaging and image analysis

After AFM imaging, fixed and stained vertebral discs were cleared and imaged with a Zeiss Axio Observer Z1 microscope equipped with a Yokogawa CSU-X1A5000 spinning disc confocal scanning unit to acquire fluorescence images of the nuclei. For optical clearing, spheroids were placed in ibidi IMM mounting medium (ibidi, Munich, Germany) whose refractive index (RI) was measured using a refractometer: 𝑛≈1.445. A 20× (NA 0.4, air) objective was used for imaging, and slices were recorded at intervals of 20 µm. Images were acquired with a Hamamatsu Orca Flash 4.0 camera. Gamma values were corrected to obtain high contrast images. Additional vertebral discs prepared for AFM measurement were also fixed and used for confocal imaging.

Image stacks acquired from the confocal microscope were analyzed using a custom-written particle detection script in Matlab. The number of nuclei per area was counted from a user-defined center position of the vertebral disc. Cells were binned in concentric rings with a width of 50 µm. Cell counts were first averaged within a bin across all image slices obtained from one animal, and then the values from different animals were averaged to determine the average phenotype.

Supplementary Text

Association of variations in the human *GPR133/ADGRD1* gene and bone phenotypes

In addition to the GWAS (Genome-Wide Association Study) and eQTL (Expression Quantitative Trait Locus) findings in mice^5^ that link GPR133/ADGRD1 function to bone mineral density, there is supporting human data. Notably, in the mouse study by Sabik et al.^5^, supplementary Table S1 references a GWAS publication in humans^6^ where the SNP rs1195932 in *GPR133/ADGRD1* was associated with bone mineral density in female probands. Reviewing the available literature and data collections, we identified three independent SNP association studies that link ADGRD1 variants with bone mineral density in humans:

- **Heel bone mineral density**: rs1880842 (chr12:131137452)^7,8^
- **Bone mineral density**: rs4759545 (chr12:131134724)^9^

All these SNPs are located in non-coding regions of *GPR133/ADGRD1* (easily visualized using the Splice-O-Mat^10^). Furthermore, several other bone mineral density-associated phenotypes are linked to SNPs within the human *GPR133/ADGRD1* locus:

- **Idiopathic scoliosis**: rs11061315 (chr12:131079007)^11^
- **Body height**: rs7957882 (chr12:131132872)^9^, rs1569019 (chr12:131091646)^12^, rs7135850 (chr12:130999445)^13^, rs7957882 (chr12:131132872)^13^, rs4075564 (chr12:131151561)^13^
- **Blood phosphate levels**: rs4759828 (chr12:131031419), rs3847687 (chr12:131040508), rs10848286 (chr12:131110975)^14^

These SNPs are also located in non-coding regions of *GPR133/ADGRD1*, preventing functional evaluation. However, both inactivating (associated with decreased bone mineral density) and activating missense mutations (potentially linked to increased bone mineral density) have been observed within the human population^15^.

References

1. Renaud, G., Stenzel, U. & Kelso, J. leeHom: adaptor trimming and merging for Illumina sequencing reads. *Nucleic Acids Res* **42,** e141 (2014).

2. Flicek, P. *et al.* Ensembl 2012. *Nucleic Acids Res* **40,** D84-90 (2012).

3. Langmead, B., Trapnell, C., Pop, M. & Salzberg, S. L. Ultrafast and memory-efficient alignment of short DNA sequences to the human genome. *Genome Biol* **10,** R25 (2009).

4. Quinlan, A. R. & Hall, I. M. BEDTools: a flexible suite of utilities for comparing genomic features. *Bioinformatics* **26,** 841–842 (2010).

5. Sabik, O. L., Calabrese, G. M., Taleghani, E., Ackert-Bicknell, C. L. & Farber, C. R. Identification of a Core Module for Bone Mineral Density through the Integration of a Co-expression Network and GWAS Data. *Cell Rep* **32,** 108145 (2020).

6. Kemp, J. P. *et al.* Identification of 153 new loci associated with heel bone mineral density and functional involvement of GPC6 in osteoporosis. *Nat Genet* **49,** 1468–1475 (2017).

7. Kim, S. K. Identification of 613 new loci associated with heel bone mineral density and a polygenic risk score for bone mineral density, osteoporosis and fracture. *PLoS One* **13,** e0200785 (2018).

8. Morris, J. A. *et al.* An atlas of genetic influences on osteoporosis in humans and mice. *Nat Genet* **51,** 258–266 (2019).

9. Kichaev, G. *et al.* Leveraging Polygenic Functional Enrichment to Improve GWAS Power. *Am J Hum Genet* **104,** 65–75 (2019).

10. Kuhn, C. K. *et al.* The repertoire and structure of adhesion GPCR transcript variants assembled from publicly available deep-sequenced human samples. *Nucleic Acids Res* **52,** 3823–3836 (2024).

11. Liu, J. *et al.* The coexistence of copy number variations (CNVs) and single nucleotide polymorphisms (SNPs) at a locus can result in distorted calculations of the significance in associating SNPs to disease. *Hum Genet* **137,** 553–567 (2018).

12. Tönjes, A. *et al.* Genetic variation in GPR133 is associated with height: genome wide association study in the self-contained population of Sorbs. *Hum Mol Genet* **18,** 4662–4668 (2009).

13. Yengo, L. *et al.* A saturated map of common genetic variants associated with human height. *Nature* **610,** 704–712 (2022).

14. Sinnott-Armstrong, N. *et al.* Genetics of 35 blood and urine biomarkers in the UK Biobank. *Nat Genet* **53,** 185–194 (2021).

15. Fischer, L., Wilde, C., Schöneberg, T. & Liebscher, I. Functional relevance of naturally occurring mutations in adhesion G protein-coupled receptor ADGRD1 (GPR133). *BMC Genomics* **17,** 609 (2016).


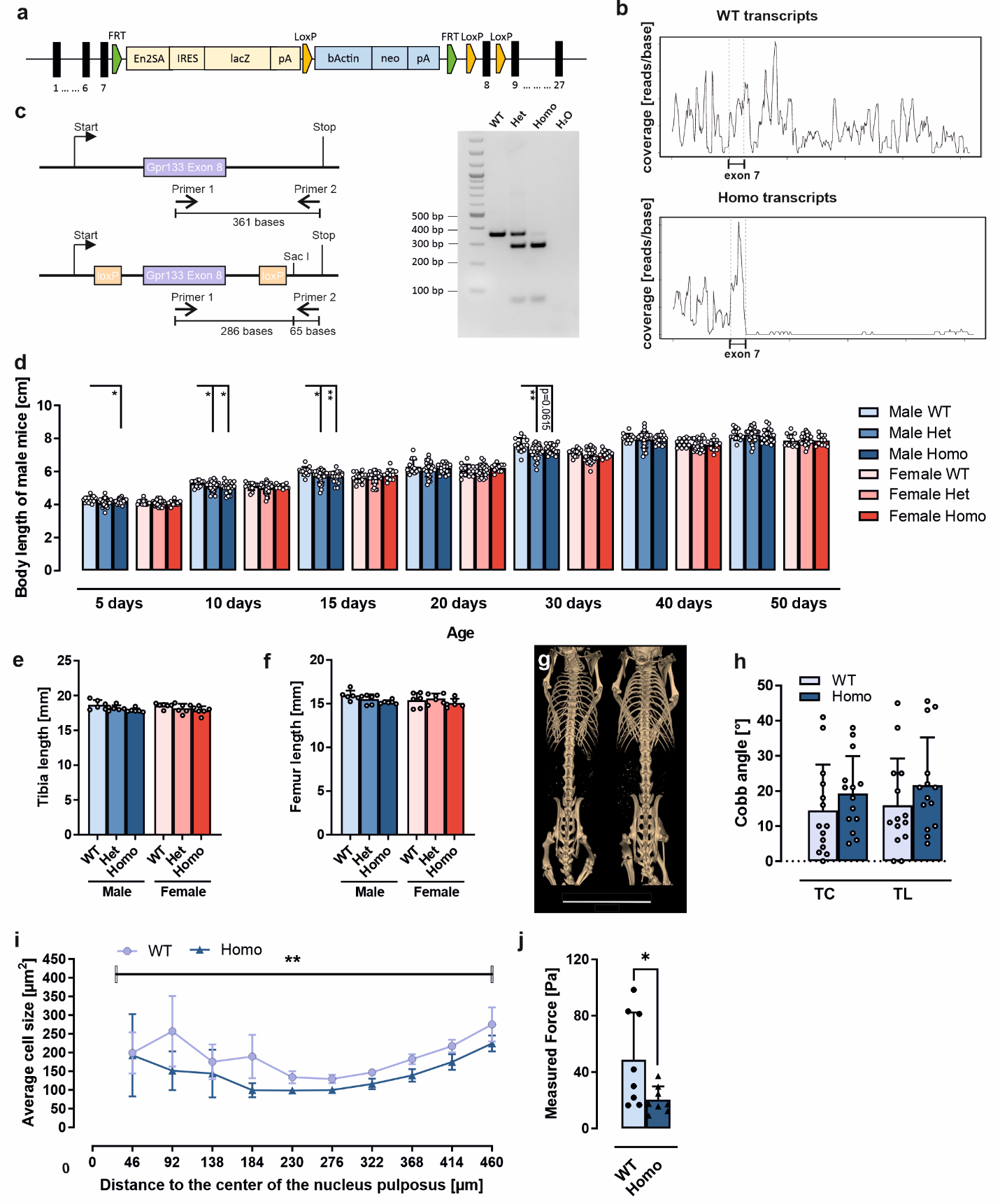


Figure. S1. *Gpr133/Adgrd1* knockout (KO) mouse design and verification.

(**a**) Genomic structure of the transgenic *Gpr133/Adgrd1* locus in mice.

(**b**) RNA-Seq analysis from mouse bladder reveals no transcript downstream of exon 7.

(**c**) Genotyping strategy for wild-type (WT), heterozygous (Het), or homozygous (Homo) *Gpr133/Adgrd1* KO mice. Insertion of a *lox*P site 3' of exon 8 introduces a recognition site for the restriction enzyme *Sac*I, resulting in a 286-bp and 65-bp fragment in the KO mice, while the WT PCR fragment remains an uncut 360-bp fragment.

(**d**) Body length of male and female WT, Het, and Homo *Gpr133/Adgrd1* KO mice between 5-50 days of age. (male: WT n = 13, Het n = 30, Homo n = 19; female: WT n = 13, Het n = 30, Homo n = 11).

(**e/f**) Femur and tibia length of 23-weeks-old male and female WT, Het, and Homo *Gpr133/Adgrd1* KO mice measured with a caliper. (n = 9 per group).

(**g**) Representative computed tomography scans of vertebrae from a WT (left) and Homo *Gpr133/Adgrd1* KO mouse (right) (both 4.5 months of age). Scale bar: 30 mm.

(**h**) Thoracocervical (TC) and thoracolumbar (TL) Cobb angles determined by x-ray imaging in WT and Homo *Gpr133/Adgrd1* KO mice (4 - 6 months of age, n=14 per group).

(**i**) Confocal microscopy analysis of vacuolated cell size within nucleus pulposi of WT and Homo *Gpr133/Adgrd1* KO mice. (6-8 months old, n = 5 per genotype).

(**j**) Atomic force measurement of the stiffness in nucleus pulposus cells from male WT and Homo *Gpr133/Adgrd1* KO mice (6-8 months old, n = 8 per genotype).

**Data information:** All quantitative data are presented as mean ± SD. Statistical analysis was performed using one-way ANOVA (d/e/f/h), Wilcoxon signed-rank test across all areas (i) or Welch test (j): *p < 0.05; **p < 0.01; ***p < 0.001 vs. WT control.


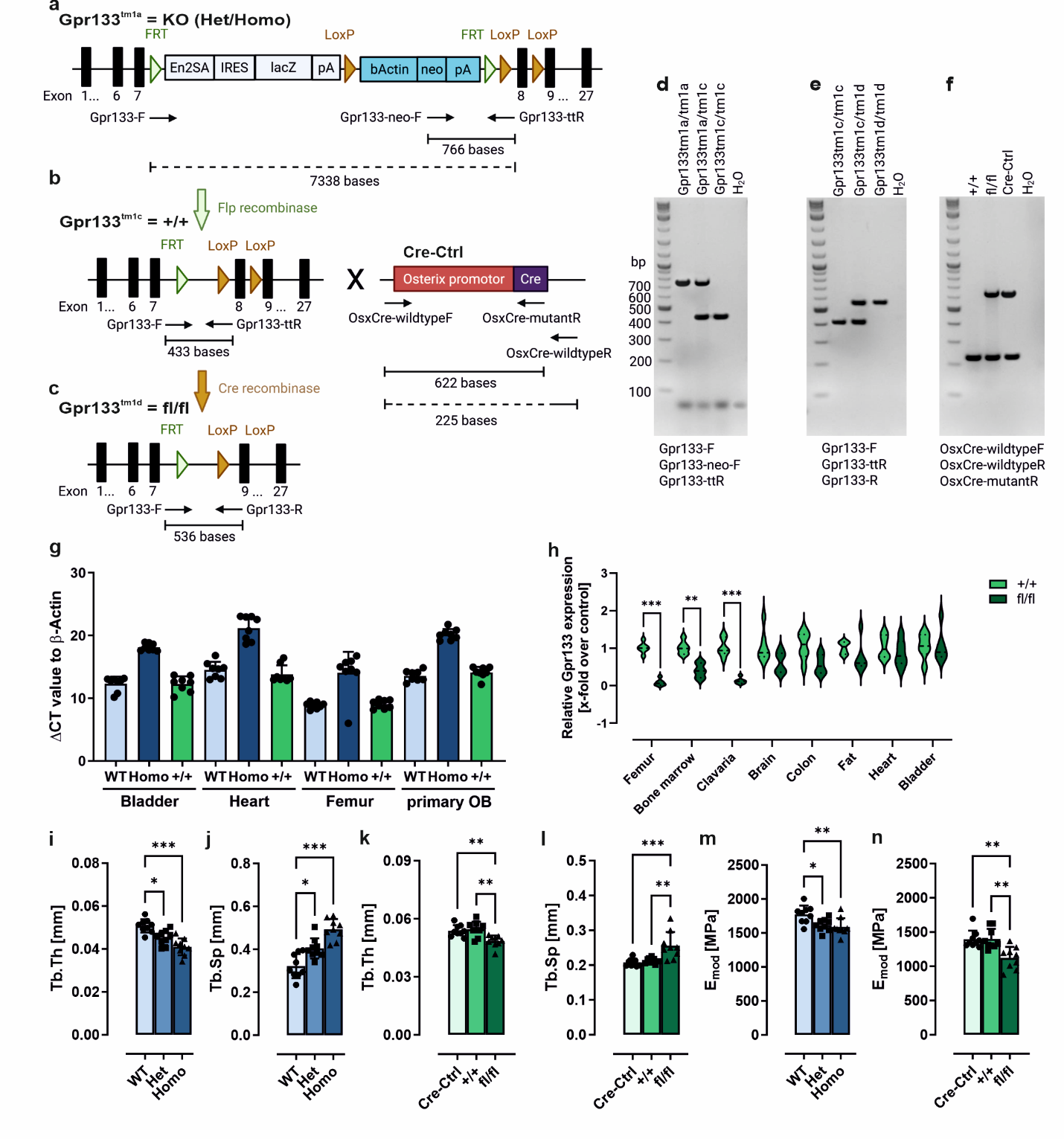


Figure. S2. Generation and genotyping of osteoblast precursor-specific *Gpr133/Adgrd1* KO mice and further bone parameters of constitutive and osteoblast-specific KO mice.

(**a**) tm1a allele (= KO (Het/Homo)): representing the targeted *Gpr133/Adgrd1*-allele containing LacZ reporter promoter-driven neo targeting cassette, FLP-FRT sites, Cre-loxP sites and *Gpr133/Adgrd1* exons (numbered 1-27).

(**b**) *Gpr133/Adgrd^tm1c^* (+/+) mice were generated by crossing *Gpr133/Adgrd ^tm1a^* mice with a Flp transgenic mouse strain, removing the FRT-flanked cassette.

(**c**) tm1d allele: the null allele representing the osteoblast precursor-specific conditional *Gpr133/Adgrd1^tm1d^* (fl/fl) mice were generated by crossing *Gpr133/Adgrd1^tm1c^* (+/+) mice with Osx-Cre recombinase transgenic mice.

(**d**) Representative image of tm1c genotyping: tm1a allele targeted band is 766 bp; tm1c allele targeted band is 433 bp.

(**e**) Representative image of tm1d genotyping: tm1d is 536 bp; tm1c band is 433 bp; heterozygotes have both bands.

(**f**) Representative image of Osx-Cre genotyping: mutant-band is 622 bp (fl/fl and Cre-control (Ctrl)) while internal control band is 225 bp. Mice who have mutant bands are Osx-Cre positive.

(**g**) Flp recombinase-mediated recombination enables the successful restoration of *Gpr133/Adgrd1* mRNA. The mRNA expression levels of *Gpr133/Adgrd1* in the urinary bladder, heart, femur, and 7-day differentiated primary osteoblasts (OB) were assessed by qPCR. These samples were collected from 23-weeks-old male WT, constitutive knockout (Homo), and Flp-recombined mice carrying the *Gpr133/Adgrd1^tm1c^* allele (functionally WT: +/+).

(**h**) *Gpr133/Adgrd1* mRNA expression relative to β-actin reference gene measured by qPCR in different tissues from 12-16-weeks-old male *Gpr133/Adgrd1^tm1c^* (+/+) and osteoblast-precursor-specific *Gpr133/Adgrd1^tm1d^* (fl/fl) mice.

(**i/j**) Femora from 23-weeks-old male WT, Het or Homo *Gpr133/Adgrd1* KO mice were examined by µCT showing changes in (**i**) trabecular thickness (Tb.Th) and (**j**) trabecular separation (Tb.Sp) in the distal femur.

(**k/l**) Femora from 23-weeks-old male Cre-Ctrl, *Gpr133/Adgrd1^tm1c^* (+/+), and osteoblast-precursor-specific *Gpr133/Adgrd1^tm1d^* (fl/fl) mice were examined by µCT showing changes in (**k**) Tb.Th and (**l**) Tb.Sp in the distal femur.

(**m/n**) A three-point bending test with elastic modulus (E_mod_) measurement was performed on femora of (**m**) constitutive and (**n**) osteoblast-specific KO mice as an indicator of bone stiffness.

**Data information:** All quantitative data are presented as mean ± SD (n = 9 per group, each dot indicates an individual mouse). (**h-n**) Statistical analysis was performed using one-way ANOVA: *p < 0.05; **p < 0.01; ***p < 0.001 comparing Het/ Homo vs. WT and fl/fl vs Cre-Ctrl or +/+.

**
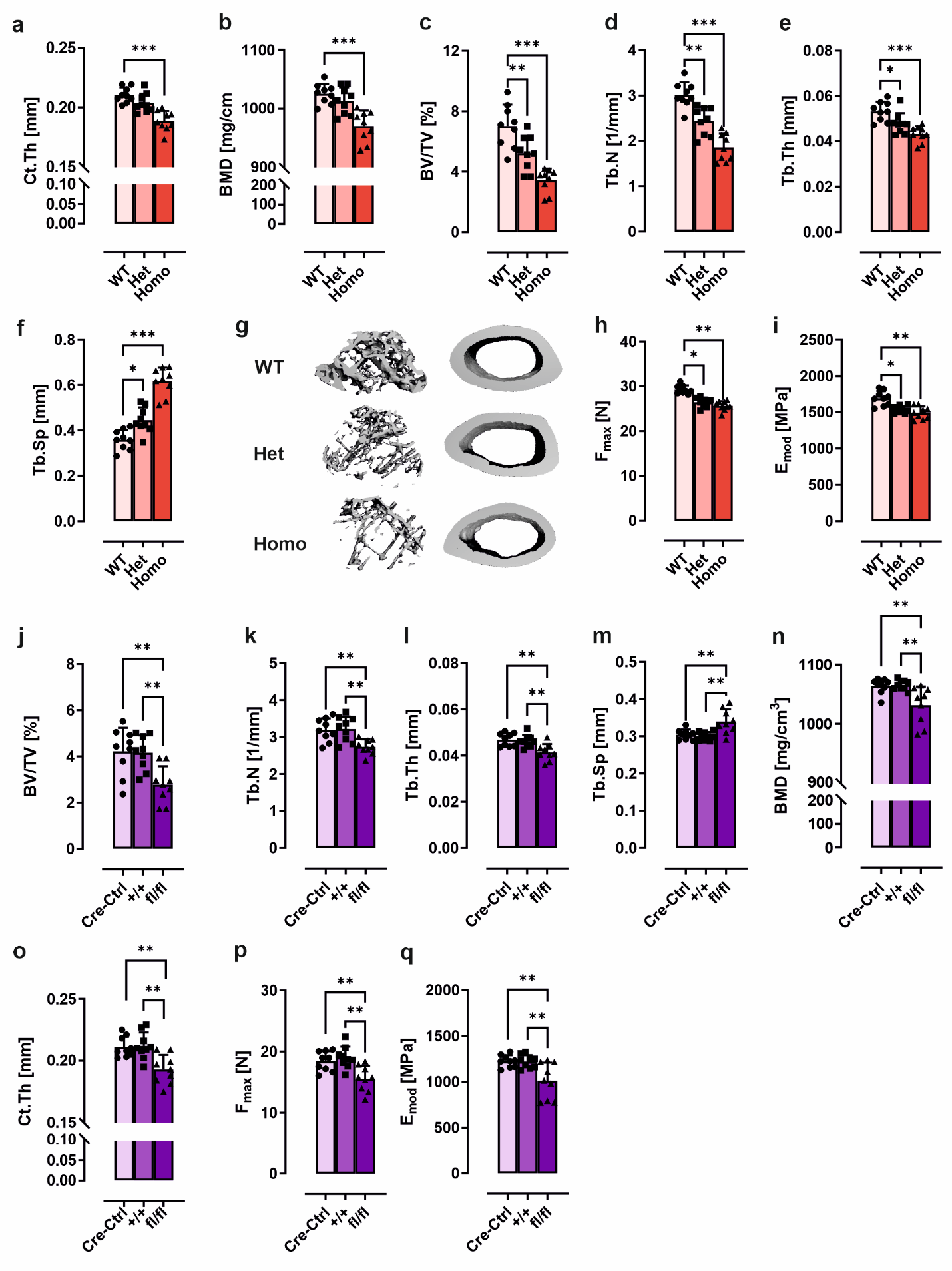
Figure. S3. Constitutive and osteoblast-specific *Gpr133/Adgrd1* deletion results in trabecular and cortical bone loss as well as reduction in bone strength and stiffness in female mice.**

(**a-f):** Femora from 23-weeks-old female WT, heterozygous (Het), or homozygous (Homo) *Gpr133/Adgrd1* KO mice were examined by µCT. **(a)** cortical thickness (Ct.Th) and (**b**) cortical bone mineral density (BMD) were measured at the femoral midshaft. (**c**) Bone volume/total volume (BV/TV), (**d**) trabecular number (Tb.N), (**e**) trabecular thickness (Tb.Th), and (**f**) trabecular separation (Tb.Sp) were assessed in the distal femur.

(**g**) Representative 3D reconstructions of the trabecular compartment and the femoral midshaft from female WT, Het, and Homo KO mice.

(**h**) The maximal load (F_max_) and (**i**) elastic modulus (E_mod_) were determined by three-point bending testing of femora from 23-weeks-old female WT, Het, or Homo *Gpr133/Adgrd1* KO mice as indicators of bone strength and stiffness, respectively.

(**j-o**) Femora from 23-weeks-old female Cre-Ctrl, *Gpr133/Adgrd1^tm1c^* (+/+) and osteoblast-precursor-specific *Gpr133/Adgrd1^tm1d^* (fl/fl) mice were examined by µCT. (**j**) BV/TV, (**k**) Tb.N, (**l**) Tb.Th, and (**m**) Tb.Sp were assessed in the distal femur. (**n**) Cortical BMD and (**o**) Ct.Th were measured at the femoral midshaft.

(**p**) F_max_ and (**q**) E_mod_ were determined by three-point bending testing of femora from 23-weeks-old female Cre-Ctrl, +/+ and fl/fl mice, serving as indicators of bone strength and stiffness, respectively.

**Data information:** All quantitative data are presented as mean ± SD (n = 9 per group, each dot indicates an individual mouse). Statistical analysis was performed by one-way ANOVA. *p < 0.05; **p < 0.01; ***p < 0.001 comparing Het/ Homo vs. WT and fl/fl vs Cre-Ctrl or +/+.


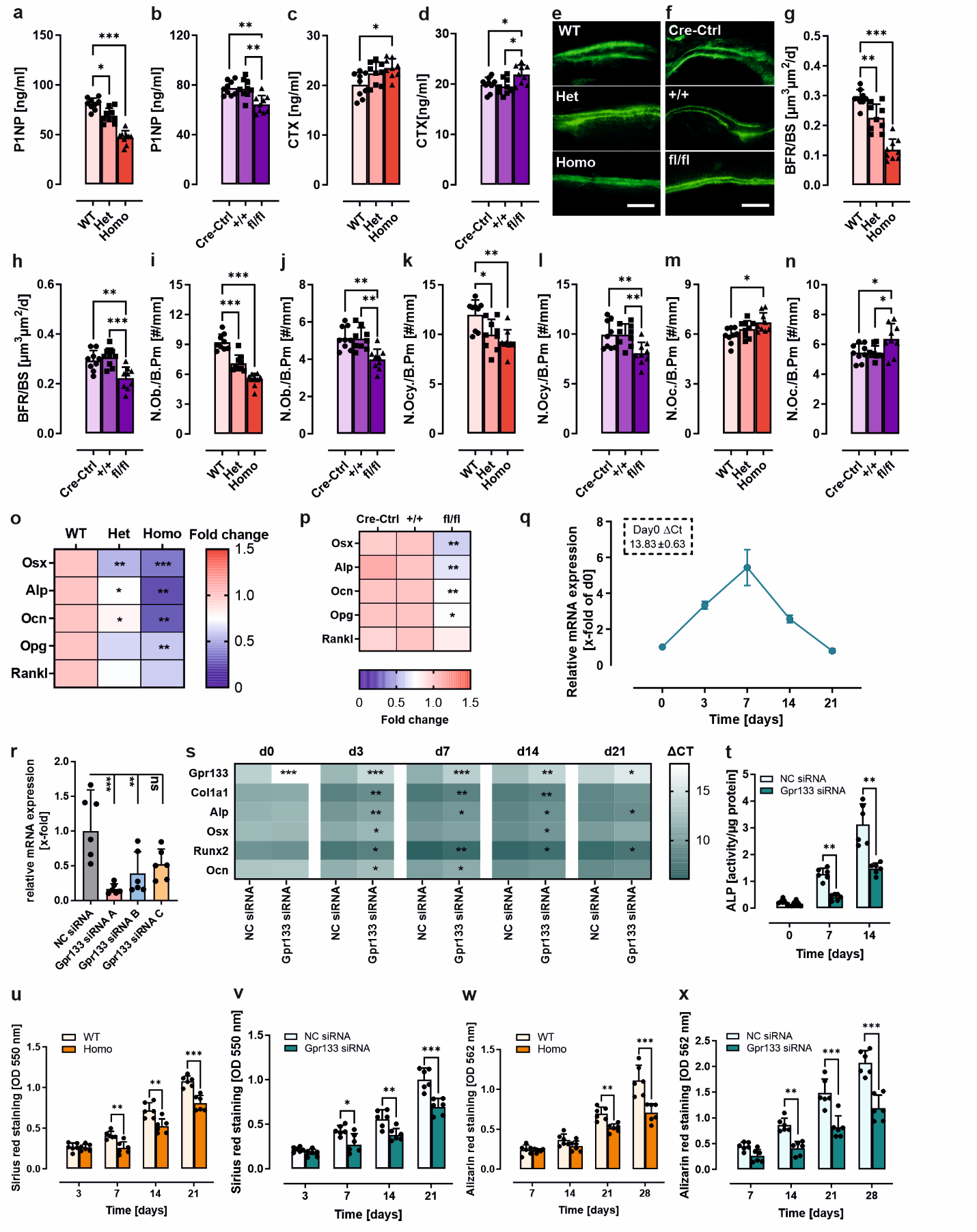


**Figure. S4. Constitutive and osteoblast precursor-specific *Gpr133/Adgrd1* deletion increases bone turnover, alters bone cell composition and reduces osteoblast differentiation and function in female mice.**

**(a-p)** 23-weeks-old female wild-type (WT), heterozygous (Het), homozygous (Homo) *Gpr133/Adgrd1* KO and Cre-control (Cre-Ctrl), *Gpr133/Adgrd1^tm1c^* (+/+) and osteoblast-precursor-specific *Gpr133/Adgrd1^tm1d^* KO (fl/fl) mice were examined.

(**a/b**) Serum concentrations of the bone formation marker type 1 procollagen amino-terminal propeptide (P1NP) and (**c, d**) the bone resorption marker cross-linked C-telopeptide of type I collagen (CTX) were measured by ELISA.

(**e/f**) Representative fluorescence images of calcein double labels. Scale bar = 10 μm. (**g, h**) Bone formation rate per bone surface (BFR/BS) was assessed by calcein double labeling of tibial bone slides and compared between groups.

(**i/j**) Number of osteoblasts per bone perimeter (N.Ob./B.pm), (**k, l**) number of osteocytes per bone perimeter (N.Ocy./B.pm), and (**m, n**) number of osteoclasts per bone perimeter (N.Oc./B.pm) were determined by TRAP staining of femoral bone slides. (**o**) Heat map of mRNA expression levels (fold change relative to WT mice) of osteoblast differentiation markers osterix (*Osx*), alkaline phosphatase (*Alp*), osteocalcin (*Ocn*), and the coupling factors osteoprotegerin (*Opg*) and receptor activator of NF-κB ligand (*Rankl*) in the tibia of WT, Het, and Homo *Gpr133/Adgrd1* KO mice or (**p**) Cre-Ctrl, +/+, and fl/fl mice. (n = 9 per group, each dot represents an individual mouse.)

(**q**) *Gpr133/Adgrd1* mRNA expression during osteoblast differentiation in MC3T3 cells was analyzed by qPCR. Results were calculated using the ΔΔCT method and normalized to β-actin mRNA, with x-fold changes shown relative to day 0 (d0). (n = 5)

(**r**) Evaluation of 3 different *Gpr133/Adgrd1* siRNAs provided by Origene showed most efficient knockdown (KD) for siRNA A, which we chose for further KD experiments. (n = 6)

(**s**) Using real-time PCR analysis, mRNA expression of *Gpr133/Adgrd1,* collagen type I alpha 1 (*Col1a1*), *Alp*, runt-related transcription factor 2 (*Runx2*), *Osx*, and *Ocn* were measured in negative control- (NC) and *Gpr133/Adgrd1* siRNA-transfected MC3T3 cells at days 0, 3, 7, 14, and 21 of differentiation. Results were calculated using the ΔCT method and normalized to β-actin mRNA. (n = 6 per group)

(**t**) ALP activity was assessed at days 0, 7, and 14 of differentiation. (n = 6 per group)

(**u**) Collagen secretion in primary WT and Homo KO osteoblasts (each dot represents an individual mouse) and (**v**) NC- and *Gpr133/Adgrd1* siRNA-transfected MC3T3 cells was determined by Sirius red staining at days 7, 14, 21, and 28 of differentiation. (n = 6 per group)

(**w**) Mineralization capacity of primary WT and Homo KO osteoblasts (each dot represents an individual mouse) and (**x**) NC- and *Gpr133/Adgrd1* siRNA-transfected MC3T3 cells was determined using Alizarin red staining at days 7, 14, 21, and 28 of differentiation. (n = 6 per group)

**Data information:** All quantitative data are presented as mean ± SD. Statistical analysis was performed using either (a-n, r) one-way ANOVA or (s-x) two-way ANOVA: * p  < 0.05; ** p  < 0.01; *** p  < 0.001 comparing Het/ Homo vs. WT, fl/fl vs Cre-Ctrl or +/+ and KD vs NC siRNA.


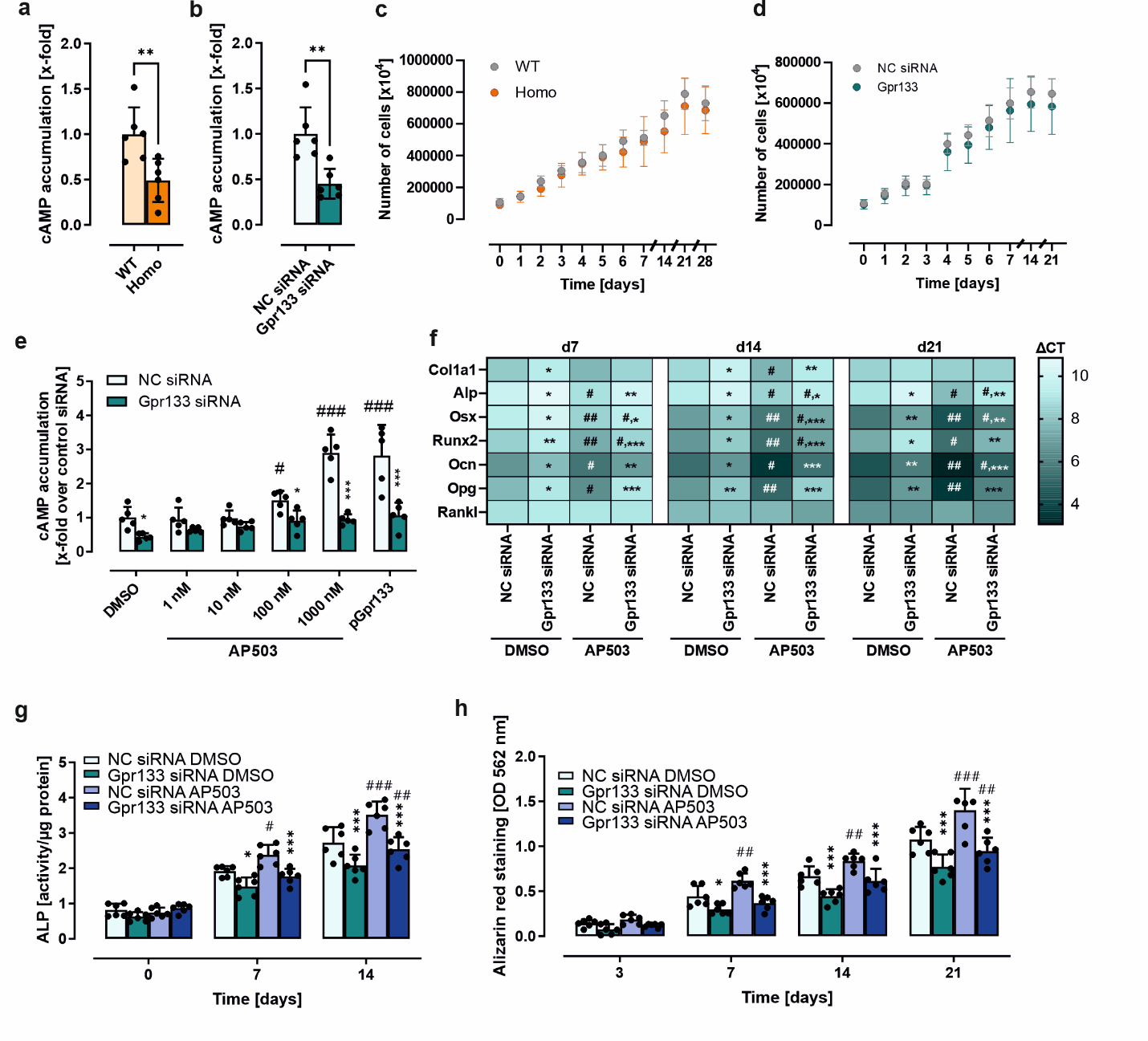


**Figure. S5. Knockdown (KD) of *Gpr133/Adgrd1* in MC3T3 cells impairs osteoblast (OB) differentiation and activity.**

(**a-b**) cAMP accumulation was measured in 7-day differentiated (**a**) primary bone marrow mesenchymal stem cells (BM-MSCs) derived from wild-type (WT) and homozygous (Homo) *Gpr133/Adgrd1* KO mice or (**b**) negative control (NC)- and *Gpr133/Adgrd1* siRNA-transfected MC3T3 cells. (n = 6 per group)

(**c/d**) Quantification of cell number in (**c**) WT and Homo *Gpr133/Adgrd1* KO primary OB as well as (**d**) NC- and *Gpr133/Adgrd1* siRNA-transfected MC3T3 cells by Hoechst staining in the course of differentiation. (n = 6 per group)

(**e**) cAMP accumulation in 7-day differentiated NC- and *Gpr133/Adgrd1* siRNA-transfected MC3T3 cells. Cells were treated with different concentrations of AP503 or 1 mM *Stachel*-derived peptide pGPR133. (n = 6 per group)

(**f**) Using real-time PCR analysis, mRNA expression of collagen type I alpha 1 (*Col1a1*), alkaline phosphatase (*Alp*), runt-related transcription factor 2 (*Runx2*), osterix (*Osx*), osteocalcin (*Ocn*), osteoprotegerin (*OPG*) and receptor activator of NF-κB ligand (*Rankl*) were measured in NC and *Gpr133/Adgrd1* siRNA-transfected MC3T3 cells at day 7, 14 and 21 of differentiation. Results were calculated based on the ΔCT method, normalized to β-actin mRNA. (n = 6 per group)

(**g**) ALP activity was assessed in NC and *Gpr133/Adgrd1* siRNA-transfected MC3T3 cells at day 0, 7 and 14 of differentiation. (n = 6 per group)

(**h**) Mineralization capacity of osteoblasts was determined in NC and *Gpr133/Adgrd1* siRNA-transfected MC3T3 cells by Alizarin red staining at day 3, 7, 14, and 21 of differentiation. (n = 6 per group)

**Data information:** Data are represented as mean ± SD. Statistical analysis was performed using two-way ANOVA: *p < 0.05; **p < 0.01; ***p < 0.001 Homo KO/ KD vs. WT/ NC siRNA, and ^#^p < 0.05; ^##^p < 0.01; ^###^p < 0.001 basal vs stimulated.


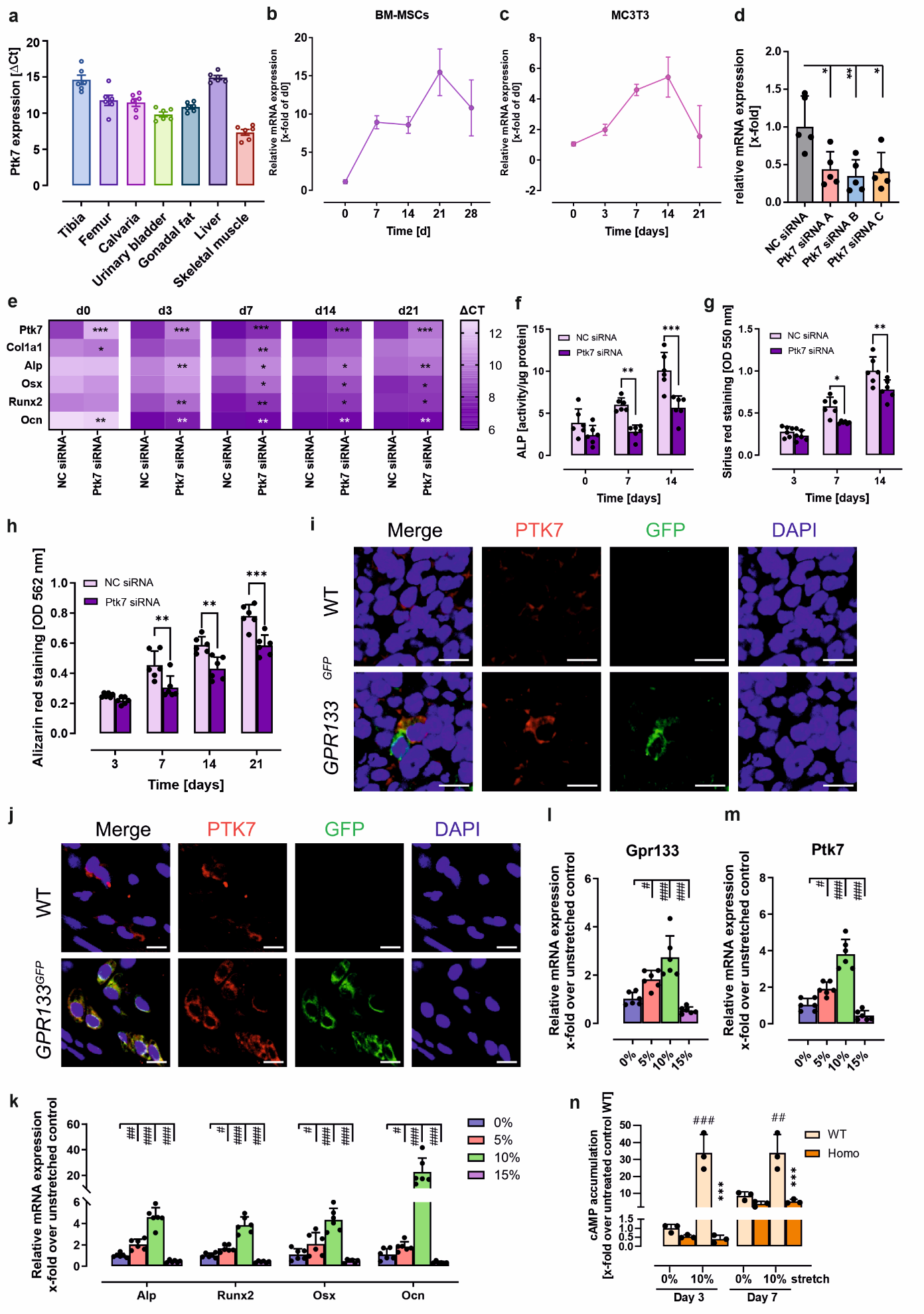


**Figure. S6. Influence of *Ptk7* and stretch on osteoblast function alone and in combination with GPR133/ADGRD1.**

(**a**) *Ptk7* mRNA expression was determined in different organs and bones of 23-weeks-old male WT mice using qPCR. (n = 6 per organ, each dot represents an individual mouse)

(**b/c**) *Ptk7* mRNA expression during osteoblast differentiation of **(b)** primary bone marrow mesenchymal stem cells (BM-MSCs) from wild-type (WT) and **(c)** MC3T3 cells was analyzed by qPCR. Results were calculated based on the ΔΔCT method, normalized to β-actin mRNA, shown are x-fold changes compared to d0. (n = 6 per cell type)

(**d**) Evaluation of 3 different *PTK7* siRNAs provided by Origene showed most efficient knockdown (KD) for siRNA B, which we chose for further KD experiments. (n = 6)

(**e**) Using real-time PCR analysis, mRNA expression of collagen type I alpha 1 (*Col1a1*), alkaline phosphatase (*Alp*), runt-related transcription factor 2 (*Runx2*), osterix (*Osx*), and osteocalcin (*Ocn*) were measured in negative control (NC)- and *Ptk7* siRNA-transfected MC3T3 cells at day 0, 3, 7, 14 and 21 of differentiation. Results were calculated based on the ΔCT method, normalized to β-actin mRNA. (n = 6 per group)

(**f**) ALP activity in NC- and *Ptk7* siRNA-transfected MC3T3 cells was assessed at day 0, 7 and 14 of differentiation. (n = 6 per group)

(**g**) Collagen secretion in NC- and *Ptk7* siRNA-transfected MC3T3 cells was determined by Sirius red staining at day 3, 7 and 14 of differentiation. (n = 6 per group)

(**h**) Mineralization capacity in NC- and *Ptk7* siRNA-transfected MC3T3 cells was determined by Alizarin red staining at day 3 7, 14, and 21 of differentiation. (n = 6 per group)

(**i/j**) Co-expression of PTK7 and GPR133/ADGRD1 in femurs of GPR133^GFP^ knock-in mice shows interaction in (**i**) *trans* and (**j**) *cis.* Scale bar: 10 µM.

(**k-m**) MC3T3 cells were differentiated for 7 days and then loaded for 8 h with (5%, 10%, 15%) or without (0%) cyclic tensile strain at the indicated percent elongation at 0.1 Hz. (**k**) Using real-time PCR analysis, mRNA expression of *Alp, Runx2,* *Osx,* and *Ocn* were assessed at day 7 of differentiation. (**l**) *Gpr133/Adgrd1* and (**m**) *Ptk7* mRNA expression were determined. Results were calculated based on the ΔΔCT method, normalized to β-actin mRNA, shown are the x-fold changes compared to unstretched controls. (n = 6 per group)

(**n**) cAMP accumulation in 3- and 7-day differentiated primary BM-MSCs derived from WT and homozygous (Homo) *Gpr133/Adgrd1* KO mice subjected to 8 h of 10% or 0% cyclic tensile strain. (n = 3 per group, each dot represents an individual mouse)

**Data information:** Data are presented as the mean ± SD. Statistical analysis was performed using (d, f-h) Student’s t test or (e, k-n) two-way ANOVA: *p < 0.05; **p < 0.01; ***p < 0.001 as indicated or compared between static and stretch, ^#^p < 0.05; ^##^p < 0.01; ^###^p < 0.001 stretched vs. unstretched control.


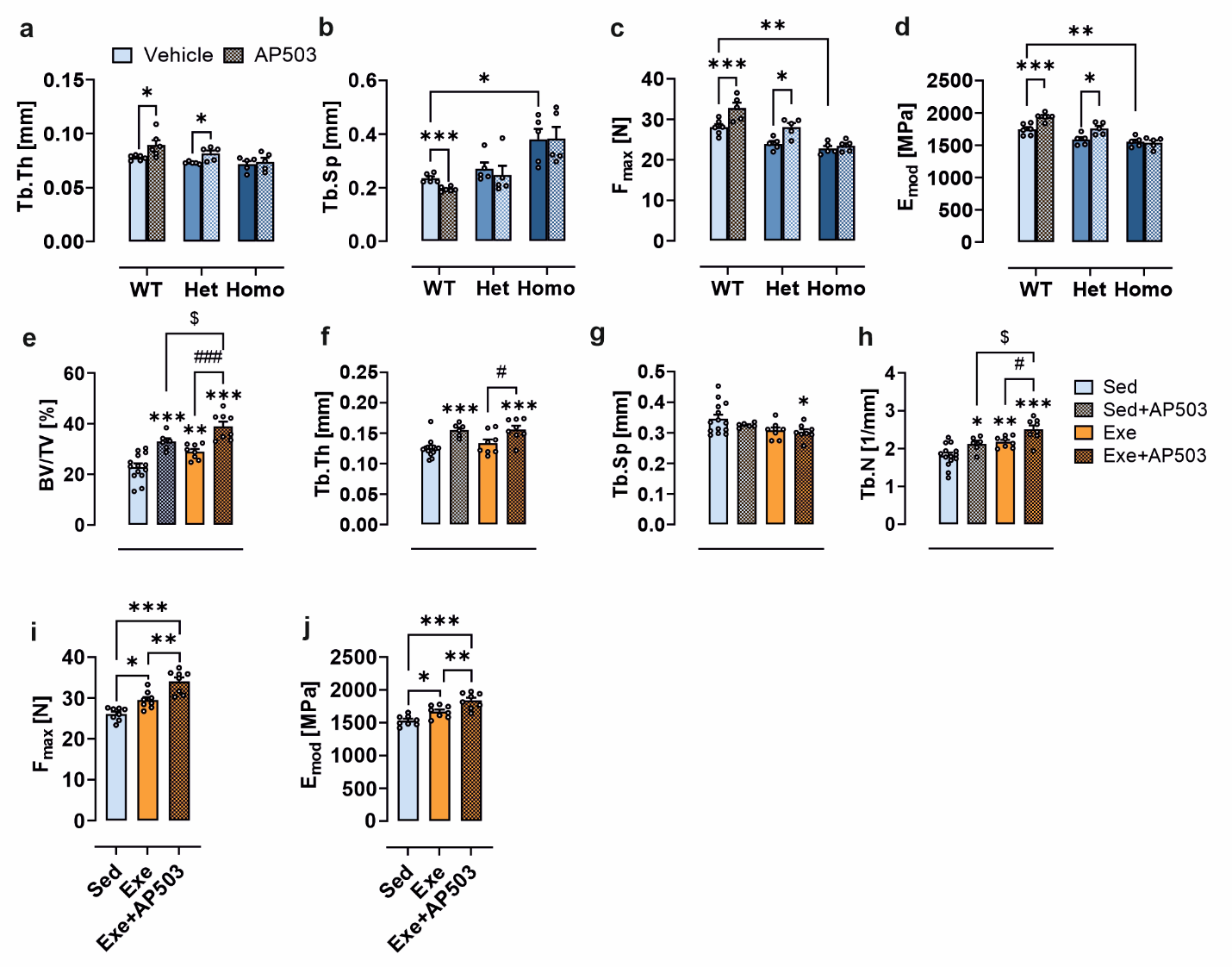


**Figure. S7. AP503 increases bone mass in male mice via GPR133/ADGRD1 and synergizes with exercise to increase bone mass in mice.**

(**a-d**) Femora from 9-weeks-old male wild-type (WT), heterozygous (Het), homozygous (Homo) *Gpr133/Adgrd1* KO mice were examined. From an age of 5-weeks-old on mice were intraperitoneally injected with vehicle or AP503 (2 mg/kg) every day for 4 weeks. (**a**) Trabecular thickness (Tb.Th) and (**b**) trabecular separation (Tb.Sp) were assessed in the distal femur through μCT. (**c/d**) A three-point bending test was performed on femora as an indicator of bone strength and stiffness. (**c**) The maximum load (Fmax) and (**d**) elastic modulus (Emod) were determined, respectively.

(**e-h**) Femora from 9-weeks-old male mice were examined through μCT. (**e**) Bone volume/total volume (BV/TV), (**f**) trabecular number (Tb.N), (**g**) trabecular thickness (Tb.Th) and (**h**) trabecular separation (Tb.Sp) were assessed in the distal femur in male WT mice following exercise and/or AP503 treatment.

(**i/j**) A three-point bending test was performed on femora as an indicator of bone strength and stiffness from WT, Het and Homo KO *Gpr133/Adgrd1* mice following exercise and/or AP503 treatment. (**i**) The maximum load (F_max_) and (**j**) elastic modulus (E_mod_) were determined, respectively.

**Data information:** Data are presented as the mean ± SEM values, n= 6-14 mice per group. Each dot represents an individual mouse. The data were analyzed via one-way ANOVA with Tukey’s test. *p < 0.05; **p < 0.01; ***p < 0.001, ^#^p < 0.05; ^##^p < 0.01; ^###^p < 0.001, ^$^p < 0.05; ns, no significant difference.


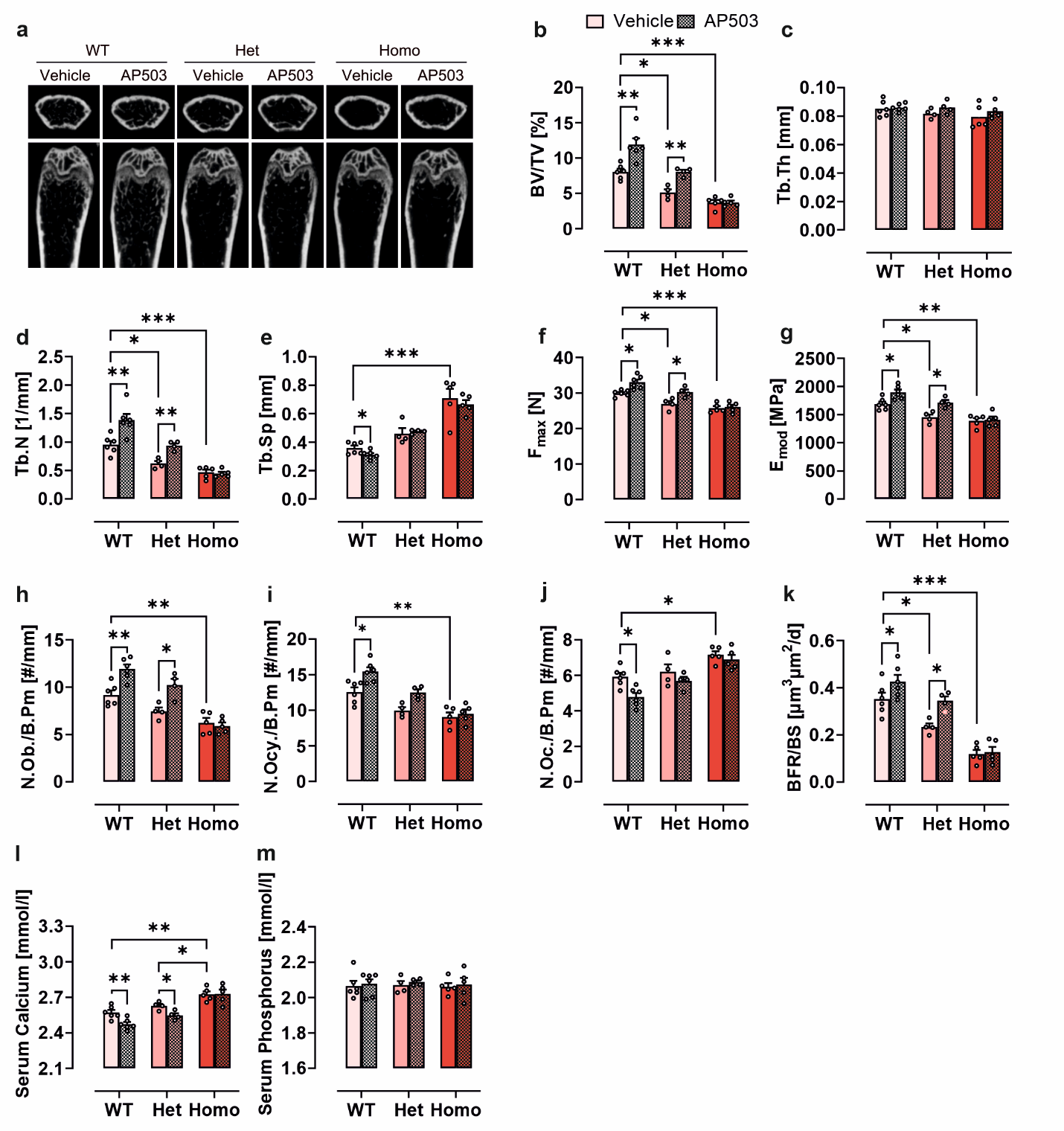


**Figure. S8. AP503 increases bone mass in female mice via GPR133/ADGRD1.**

Femora from 9-weeks-old female wild-type (WT), heterozygous (Het) or homozygous (Homo) *Gpr133/Adgrd1* KO mice were examined. 5-weeks-old WT, Het or Homo mice were intraperitoneally injected with vehicle or AP503 (2 mg/kg) every day for 4 weeks.

(**a**) Representative μCT images of distal femurs (cross sections and longitudinal sections). (**b**) Bone volume/total volume (BV/TV), (**c**) trabecular thickness (Tb.Th), (**d**) trabecular number (Tb.N), and (**e**) trabecular separation (Tb.Sp) were assessed in the distal femur.

(**f/g**) A three-point bending test was performed on femora as an indicator of bone strength and stiffness. (**f**) The maximum load (F_max_) and (**g**) elastic modulus (E_mod_) were determined, respectively.

(**h-j**) Number of (**h**) osteoblasts (N.Ob./B.pm), (**i**) osteocytes (N.Ocy./B.pm), and (**j**) number of osteoclasts (N.Oc./B.pm) were determined by Tartrate-resistant acid phosphatase (TRAP) staining of femoral bone slides.

(**k**) Bone formation rate per bone surface (BFR/BS) was assessed by calcein double labeling of tibial bone slides and compared between groups.

(**l**) Serum calcium and (**m**) phosphate concentrations from control and AP503 treated female WT, Het or Homo *Gpr133/Adgrd1* KO mice.

**Data information:** Data are presented as the mean ± SEM values, n=4-6 mice per group. Each dot indicates an individual mouse. The data were analyzed via one-way ANOVA with Tukey’s test. *p < 0.05, **p < 0.01, ***p < 0.001; ns, no significant difference.

Table S1. Bone phenotype of the fourth lumbar vertebral body from 23-weeks-old male and female constitutive wild-type (WT), heterozygous (Het) or homozygous (Homo) *Gpr133/Adgrd1* KO mice.

|  | **WT** | **Het** | **Homo** |
| --- | --- | --- | --- |
| **Male** | **N = 9** | **N = 9** | **N = 9** |
| *µCT* |  |  |  |
| BVTV [%] | 10.83 ± 1.62 | 8.70 ± 1.50** | 6.62 ± 1.06*** |
| Tb.Th [µm] | 52.66 ± 1.63 | 47.04 ± 3.17** | 41.61 ± 1.50*** |
| Tb.N [1/mm] | 2.99 ± 0.30 | 2.71 ± 0.30* | 2.27 ± 0.13*** |
| Tb.Sp [mm] | 0.35 ± 0.04 | 0.41 ± 0.04* | 0.48 ± 0.05*** |
| *Compression test* |  |  |  |
| F_max_ [N] | 53.10 ± 6.93 | 42.44 ± 6.17* | 41.14 ± 7.82** |
| E_mod_ [MPa] | 871.11 ± 150.45 | 639.61 ± 136.68* | 594.54 ± 139.14** |
| **Female** | **N = 9** | **N = 9** | **N = 9** |
| *µCT* |  |  |  |
| BVTV [%] | 8.16 ± 0.68 | 6.19 ± 0.55** | 4.54 ± 0.83*** |
| Tb.Th [µm] | 41.7 ± 3.61 | 36.1 ± 4.62** | 32.9 ± 2.56*** |
| Tb.N [1/mm] | 2.84 ± 0.17 | 2.48 ± 0.13** | 2.12 ± 0.15*** |
| Tb.Sp [mm] | 0.32 ± 0.03 | 0.39 ± 0.02** | 0.48 ± 0.04*** |
| *Compression test* |  |  |  |
| F_max_ [N] | 52.06 ± 4.27 | 40.94 ± 7.12* | 39.87 ± 6.14** |
| E_mod_ [Mpa] | 858.70 ± 132.30 | 639.30 ± 146.63* | 577.29 ± 95.11** |

BV/TV = bone volume/trabecular volume, Tb.Th = trabecular thickness, Tb.N = trabecular number, Tb.Sp = trabecular separation, F_max_ = maximal force, E_mod_ = elastic modulus. Data represent the mean ± SD. Statistical analysis was performed using one-way ANOVA. *p < 0.05, **p < 0.01, ***p < 0.001 Homo/Het vs WT control

**Table S2. Bone phenotype of fourth lumbar vertebral body from 23-weeks-old male and female osteoblast precursor-specific *Gpr133/Adgrd1* KO and control mice.**

|  | **Cre-Ctrl** | **+/+** | **fl/fl** |
| --- | --- | --- | --- |
| **Male** | **N = 9** | **N = 9** | **N = 9** |
| *µCT* |  |  |  |
| BVTV [%] | 24.08 ± 2.01 | 24.29 ± 2.31 | 18.49 ± 2.64^##^;** |
| Tb.Th [µm] | 62.51 ± 3.78 | 61.00 ± 3.02 | 52.29 ± 7.03^##^,* |
| Tb.N [1/mm] | 4.55 ± 0.14 | 4.52 ± 0.17 | 3.91 ± 0.23^##^,** |
| Tb.Sp [mm] | 0.22 ± 0.01 | 0.23 ± 0.02* | 0.28 ± 0.04^#^;* |
| *Compression test* |  |  |  |
| F_max_ [N] | 340.54 ± 43.99 | 342.15 ± 32.36 | 244.77 ± 76.06^##^,** |
| E_mod_ [Mpa] | 1016.61 ± 113.51 | 1007.02 ± 78.16 | 743.59 ± 243.45^##^,** |
| **Female** | **N = 9** | **N = 9** | **N = 9** |
| *µCT* |  |  |  |
| BVTV [%] | 21.61 ± 2.19 | 22.36 ± 3.12 | 17.21 ± 3.17^##^,** |
| Tb.Th [µm] | 54.51 ± 2.02 | 54.47 ± 3.03 | 50,10 ± 2.85^##^,** |
| Tb.N [1/mm] | 4.55 ± 0.14 | 4.52 ± 0.17 | 3.92 ± 0.23^##^,** |
| Tb.Sp [mm] | 0.22 ± 0.01 | 0.23 ± 0.02 | 0.28 ± 0.04^##^,** |
| *Compression test* |  |  |  |
| F_max_ [N] | 289.51 ± 28.71 | 299.16 ± 40.21 | 234.15 ± 34.69^##^,** |
| E_mod_ [Mpa] | 1078.62 ± 59.77 | 1100.88 ± 71.83 | 934.80 ± 125.11^##^,** |

BV/TV = bone volume/trabecular volume, Tb.Th = trabecular thickness, Tb.N = trabecular number, Tb.Sp = trabecular separation, F_max_ = maximal force, E_mod_ = elastic modulus. Data represent the mean ± SD. Statistical analysis was performed using one-way ANOVA. ^#^p < 0.05, ^##^p < 0.01, ^###^p < 0.001 fl/fl versus Cre-Ctrl; *p < 0.05, **p < 0.01, ***p < 0.001 fl/fl vs +/+ control.

Table S3. Histological analysis of tibiae from 23-weeks-old male and female constitutive and osteoblast precursor-specific *Gpr133/Adgrd1* KO and control mice.

| **Constitutive KO** | **WT** | **Het** | **Homo** |
| --- | --- | --- | --- |
| **Male** | **N = 9** | **N = 9** | **N = 9** |
| MS/BS [%] | 22.49 ± 3.75 | 20.09 ± 4.11* | 15.10 ± 3.72*** |
| MAR [µm/d] | 1.22±0.13 | 1.02±0.13* | 0.95±0.13*** |
|  |  |  |  |
| **Female** | **N = 9** | **N = 9** | **N = 9** |
| MS/BS [%] | 28.99 ± 3.36 | 27.39 ± 4.81* | 17.92 ± 4.63*** |
| MAR [µm/d] | 1.01 ± 0.05 | 0.83 ± 0.10* | 0.68 ± 0.16*** |
| **osteoblast precursor-specific KO** | **Cre-Ctrl** | **+/+** | **fl/fl** |
| **Male** | **N = 9** | **N = 9** | **N = 9** |
| MS/BS [%] | 27.61 ± 2.96 | 27.76 ± 3.12 | 22.27 ± 2.34^#^,** |
| MAR [µm/d] | 1.01 ± 0.03 | 1.02 ± 0.08 | 0.90 ± 0.08^#^,** |
|  |  |  |  |
| **Female** | **N = 9** | **N = 9** | **N = 9** |
| MS/BS [%] | 29.22 ± 3.53 | 29.23 ± 2.57 | 24.13 ± 4.44^###^;*** |
| MAR [µm/d] | 1.04 ± 0.05 | 1.02 ± 0.06 | 0.93 ± 0.07^##^,* |

MS/BS = mineralizing surface/bone surface, MAR = mineral apposition rate, Data represent the mean ± SD. Statistical analysis was performed using one-way ANOVA. For constitutive KO: *p < 0.05, **p < 0.01, ***p < 0.001 Homo/Het vs WT control or for osteoblast precursor-specific KO: ^#^p < 0.05, ^##^p < 0.01, ^###^p < 0.001 fl/fl vs Cre-Ctrl or *p < 0.05, **p < 0.01, ***p < 0.001 fl/fl vs +/+ control.

Table S4. Histological analysis of third and fourth lumbar vertebral body from 23-weeks-old male and female constitutive and osteoblast precursor-specific *Gpr133/Adgrd1* KO and control mice.

| **Constitutive KO** | **WT** | **Het** | **Homo** |
| --- | --- | --- | --- |
| **Male** | **N = 9** | **N = 9** | **N = 9** |
| MS/BS [%] | 32.33 ± 2.95 | 26.25 ± 3.54* | 24.44 ± 3.94** |
| MAR [µm/d] | 3.29 ± 0.61 | 2.49 ± 0.40* | 2.28 ± 0.56** |
| BFR [µm^3^µm^2^/d] | 1.05 ± 0.17 | 0.70 ± 0.14* | 0.67 ± 0.26** |
| N.Ob/B.Pm [#/mm] | 8.41 ± 0.64 | 7.22 ± 0.37** | 6.56 ± 0.33*** |
| N.Oc/B.Pm [#/mm] | 5.69 ± 0.43 | 6.00 ± 0.60 | 6.76 ± 0.63* |
| N.Ocy/B.Pm [#/mm] | 8.70 ± 0.81 | 7.62 ± 0.57* | 6.19 ± 0.53*** |
|  |  |  |  |
| **Female** | **N = 9** | **N = 9** | **N = 9** |
| MS/BS [%] | 29.71 ± 3.16 | 23.45 ± 4.60* | 21.54 ± 4.56** |
| MAR [µm/d] | 3.29 ± 0.61 | 2.49 ± 0.40* | 2.28 ± 0.56** |
| BFR [µm^3^µm^2^/d] | 1.19 ± 0.26 | 0.87 ± 0.20* | 0.74 ± 0.22** |
| N.Ob/B.Pm [#/mm] | 5.43 ± 0.29 | 4.61 ± 0.37* | 3.57 ± 0.51** |
| N.Oc/B.Pm [#/mm] | 6.00 ± 0.68 | 6.50 ± 0.76* | 7.08 ± 0.79* |
| N.Ocy/B.Pm [#/mm] | 9.22 ± 1.13 | 7.05 ± 1.04* | 5.80 ± 0.57*** |
| **osteoblast precursor-specific KO** | **Cre-Ctrl** | **+/+** | **fl/fl** |
| **Male** | **N = 9** | **N = 9** | **N = 9** |
| MS/BS [%] | 27.03 ± 3.68 | 27.04 ± 3.18 | 21.82 ± 2.89^###^,*** |
| MAR [µm/d] | 0.88 ± 0.08 | 0.84 ± 0.11 | 0.70 ± 0.15^##^,* |
| BFR [µm^3^µm^2^/d] | 0.24 ± 0.04 | 0.24 ± 0.03* | 0.16 ± 0.03^###^,*** |
| N.Ob/B.Pm [#/mm] | 5.34 ± 0.42 | 5.34 ± 0.47 | 4.24 ± 0.62^###^,*** |
| N.Oc/B.Pm [#/mm] | 5.43 ± 0.65 | 5.42 ± 0.92 | 6.66 ± 0.95^#^,* |
| N.Ocy/B.Pm [#/mm] | 7.06 ± 1.32 | 6.86 ± 1.36* | 5.30 ± 0.78^##^;* |
|  |  |  |  |
| **Female** | **N = 9** | **N = 9** | **N = 9** |
| MS/BS [%] | 24.55 ± 1.73 | 24.63 ± 2.22 | 20.55 ± 2.43^##^,** |
| MAR [µm/d] | 0.83 ± 0.09 | 0.84 ± 0.09 | 0.71 ± 0.08^#^,** |
| BFR [µm^3^µm^2^/d] | 0.22 ± 0.03 | 0.22 ± 0.03 | 0.16 ± 0.02^##^,** |
| N.Ob/B.Pm [#/mm] | 5.30 ± 0.37 | 5.16 ± 0.54 | 4.06 ± 0.68^###^,*** |
| N.Oc/B.Pm [#/mm] | 5.12 ± 0.61 | 5.10 ± 0.60 | 6.42 ± 1.28^#^,* |
| N.Ocy/B.Pm [#/mm] | 6.33 ± 0.74 | 6.48 ± 0.84 | 4.77 ± 0.87^##^,*** |

MS/BS = mineralizing surface/bone surface, MAR = mineral apposition rate, BFR/BS = bone formation rate/bone surface, N.Ob/B.Pm= number of osteoblasts, N.Oc/B.Pm = number of osteoclasts/bone perimeter, N.Ocy/B.Pm = number of osteocytes/ bone perimeter. Data represent the mean ± SD. Statistical analysis was performed using one-way ANOVA. For constitutive KO: *p < 0.05, **p < 0.01, ***p < 0.001 Homo/Het vs WT control or for osteoblast precursor-specific KO: ^#^p < 0.05, ^##^p < 0.01, ^###^p < 0.001 fl/fl versus Cre-Ctrl or *p < 0.05, **p < 0.01, ***p < 0.001 fl/fl vs +/+ control.

Table S5. List of primers used for qPCR.

| Gene | Forward primer (5′→3′) | Reverse Primer (5′→3′) |
| --- | --- | --- |
| *Alp* | CTACTTGTGTGGCGTGAAGG | CTGGTGGCATCTCGTTATCC |
| *Axin2* | GCAGTGATGGAGGAAAATGC | ATTCAAGGTGGGGAGGTAGC |
| *Col1a1* | CGTGACCAAAAACCAAAAGTG | GGGGTGGAGAAAGGAGCAGA |
| *Csk* | CCAGTGGGAGCTATGGAAGA | AAGTGGTTCATGGCCAGTTC |
| *Dkk1* | GCC TCC GAT CAT CAG ACG GT | GCA GGT GTG GAG CCT AGA AG |
| *Gpr133/Adgrd1* | GTCCCACTGAAGCTCACACA | CTACCAGGACAGCGAAGGAC |
| *Nfatc1* | GTTCCTTCAGCCAATCATCC | GGAGGTGATCTCGATTCTCG |
| *Ocn* | GCGCTCTGTCTCTCTGACCT | ACCTTATTGCCCTCCTGCTT |
| *Opg* | CCTTGCCCTGACCACTCTTA | ACACTGGGCTGCAATACACA |
| *Osx* | CTTCCCAATCCTATTTGCCGTTT | CGGCCAGGTTACTAACACCAATCT |
| *Ptk7* | AGAACGGTTCCCTGGTGATCCA | TCCGAGTCTTCCATCACTGGCT |
| *Rankl* | CCAAGATCTCTAACATGACG | CACCATCAGCTGAAGATAGT |
| *SOST* | CGTGCCTCATCTGCCTACTT | TGACCTCTGTGGCATCATTC |
| *Tcf* | CCAGTGCTACAGTCAAGGACAC | TGATGAGGGTGCTGAACAAC |
| *Trap* | GCAGTATCTTCAGGACGAGAAC | TCCATAGTGAAACCGCAAGTAG |
| *Wnt3a* | GAACCGTCACAACAATGAGG | GCATTCCTTGATGCCTGTCT |
| *Wnt5a* | CCAACTGGCAGGACTTTCTC | GCATTCCTTGATGCCTGTCT |
| *Β-Actin* | GCTCTTTTCCAGCCTTCCTT | CGGATGTCAACGTCACACTT |

*Alp*: alkaline phosphatase, *Col1a1*: Collagen type I alpha 1, *Dkk1*: dickkopf-1, *Gpr133*: G protein-coupled receptor 133, *Ocn*: Osteocalcin, *Opg*: Osteoprotegerin, *Osx*: osterix, *Ptk7*: protein tyrosine kinase, *Rankl*: receptor activator of NF-κB ligand, *SOST*: Sclerostin, *Tcf1/7*: transcription factor 7
